# A gene transfer event suggests a long-term partnership between eustigmatophyte algae and a novel lineage of endosymbiotic bacteria

**DOI:** 10.1038/s41396-018-0177-y

**Published:** 2018-06-07

**Authors:** Tatiana Yurchenko, Tereza Ševčíková, Pavel Přibyl, Khalid El Karkouri, Vladimír Klimeš, Raquel Amaral, Veronika Zbránková, Eunsoo Kim, Didier Raoult, Lilia M. A. Santos, Marek Eliáš

**Affiliations:** 1 Faculty of Science, Department of Biology and Ecology, Life Science Research Centre, University of Ostrava, Chittussiho 10, Ostrava, 710 00 Czech Republic; 2 Faculty of Science, Institute of Environmental Technologies, University of Ostrava, Chittussiho 10, Ostrava, 710 00 Czech Republic; 3 Centre for Phycology and Biorefinery Research Centre of Competence, Institute of Botany CAS, Dukelská 135, Třeboň, CZ-379 82 Czech Republic; 4 Unité de Recherche en Maladies Infectieuses et Tropicales Emergentes (URMITE), UM63, CNRS7278, IRD198, INSERMU1095, Institut Hospitalo-Universitaire Méditerranée-Infection, Aix-Marseille Université, Faculté de Médecine, 27 boulevard Jean Moulin, Marseille cedex 5, 13385 France; 5 Department of Life Sciences, Coimbra Collection of Algae (ACOI), University of Coimbra, Coimbra, 3000-456 Portugal; 6 Sackler Institute for Comparative Genomics, American Museum of Natural History, Central Park West at 79th Street, New York, NY 10024 USA; 7 Division of Invertebrate Zoology, American Museum of Natural History, Central Park West at 79th Street, New York, NY 10024 USA

## Abstract

Rickettsiales are obligate intracellular bacteria originally found in metazoans, but more recently recognized as widespread endosymbionts of various protists. One genus was detected also in several green algae, but reports on rickettsialean endosymbionts in other algal groups are lacking. Here we show that several distantly related eustigmatophytes (coccoid algae belonging to Ochrophyta, Stramenopiles) are infected by *Candidatus* Phycorickettsia gen. nov., a new member of the family Rickettsiaceae. The genome sequence of *Ca*. Phycorickettsia trachydisci sp. nov., an endosymbiont of *Trachydiscus minutus* CCALA 838, revealed genomic features (size, GC content, number of genes) typical for other Rickettsiales, but some unusual aspects of the gene content were noted. Specifically, *Phycorickettsia* lacks genes for several components of the respiration chain, haem biosynthesis pathway, or c-di-GMP-based signalling. On the other hand, it uniquely harbours a six-gene operon of enigmatic function that we recently reported from plastid genomes of two distantly related eustigmatophytes and from various non-rickettsialean bacteria. Strikingly, the eustigmatophyte operon is closely related to the one from *Phycorickettsia*, suggesting a gene transfer event between the endosymbiont and host lineages in early eustigmatophyte evolution. We hypothesize an important role of the operon in the physiology of *Phycorickettsia* infection and a long-term eustigmatophyte-*Phycorickettsia* coexistence.

## Introduction

Eukaryotic cells are inherently endowed with the capability of harbouring prokaryotic endosymbionts [1–3]. The list of known host–endosymbiont pairs is growing rapidly, with lesser-studied eukaryotic taxa, including protozoans, algae, and microscopic fungi, proving to be a particularly rich resource of novel endosymbiotic systems [4–8]. The nature of the endosymbiont–host relationship varies from mutualism to parasitism and from an accidental nonspecific interaction to permanent integration of the endosymbiont. Metabolic functions of prokaryotic endosymbionts underpinning their mutualistic relationship with the host include fixation of N_2_ and/or synthesis of nitrogen-containing compounds (amino acid, enzyme cofactors etc.), photosynthesis, or methanogenesis [2, 9–11]. It is conceivable that novel, unexpected forms of metabolic interactions are to be found with characterization of additional endosymbiont–host systems.

One of the most prominent groups of endosymbiotic bacteria is the order Rickettsiales, presently divided into three families: Rickettsiaceae, Anaplasmataceae, and Candidatus Midichloriaceae [12, 13]. Members of Rickettsiales were originally found in association with terrestrial animals, but subsequently they have been reported from an ever-growing list of aquatic hosts, particularly diverse protists (ciliates and amoebae). The known host range has recently been extended to several distantly related taxa of green algae [4, 14–16] harbouring bacteria representing two closely related lineages, *Candidatus* Megaira polyxenophila and *Ca*. Megaira subclade B, which are promiscuous partners of additional host types (ciliates, vamyrellids, haplosporidians, or corals) [4, 12, 17, 18]. Such a broad occurrence suggests that endosymbionts belonging to *Ca*. Megaira are loosely associated with their hosts and can be easily transferred to new, phylogenetically remote hosts.

Eustigmatophytes are a class of unicellular algae belonging to the Ochrophyta (plastid-bearing stramenopiles). They were recognized as a separate group decades ago, but for long limited attention was paid to them, partly because they were considered a species-poor taxon of little ecological significance. However, recent years have witnessed a burst of interest in eustigmatophytes, primarily driven by prospects for their biotechnological exploitation [19]. Nevertheless, the research on eustigmatophytes has been heavily biased toward a single group, the genus *Nannochloropsis* (including the recently segregated genus *Microchloropsis*; [20]), resulting in development of extensive genomic resources for most species of this clade [21–24]. Other eustigmatophytes have been investigated to a much less extent and significant gaps remain in the knowledge of the basic eustigmatophyte biology.

We have recently applied next-generation sequencing technologies to obtain the first genomic data from three algae representing eustigmatophyte lineages with different phylogenetic distance from the *Nannochloropsis*-*Microchloropsis* clade. These include *Trachydiscus minutus* representing a recently delimited clade Goniochloridales [25, 26], and *Vischeria* sp. CAUP Q 202 and *Monodopsis* sp. MarTras21 belonging to the second main eustigmatophyte clade, Eustigmatales [27]. We have previously reported sequences of organellar genomes of all three algae [27–29]. The most striking finding, directly relevant to the present study, was the identification of a novel six-gene operon in the plastid genomes of *Vischeria* and *Monodopsis* [29]. Comparative genomic and phylogenetic analyses revealed that this operon, denoted *ebo* (eustigmatophyte-bacterial operon), is widespread in different groups of bacteria and was horizontally transferred into an ancestor of *Vischeria* and *Monodopsis*. The function of this operon remains unknown, but bioinformatic analyses of the six encoded proteins revealed most of them as putative enzymes underpinning the synthesis of an unknown prenylated cyclitol or its derivative [29].

Here we report on another unexpected outcome of our genomic investigations of eustigmatophytes. Sequencing the genome of *T. minutus* surprisingly yielded genomic data from a novel Rickettsiaceae bacterium that proved to be an endosymbiont occurring more widely in eustigmatophyte algae. In addition, this new bacterial lineage turned out to be the likely donor of the eustigmatophyte *ebo* operon, pointing to a long history of co-evolution, including gene exchange, between the algal host and its bacterial partner.

## Materials and methods

Below we provide a summary on the methods employed; for further technical details see Supplementary Materials and Methods.

DNA for PCR experiments was the same as used before [26, 28] or obtained from newly isolated algal strains or strains ordered from public culture collections (Supplementary Table 1). The presence of the novel rickettsialean endosymbiont (*Ca*. Phycorickettsia) was tested by PCR amplification of 16S ribosomal DNA (rDNA) using newly designed specific primers and sequencing of the products. Sequences corresponding to *Ca*. Phycorickettsia were deposited at GenBank with accession numbers listed in Table 1. Fluorescence in situ hybridization (FISH) with tyramide signal amplification was performed to detect *Ca*. Phycorickettsia in cells of the alga *Trachydiscus minutus* CCALA 838. A horseradish peroxidase-conjugated probe designed to specifically match the 16S ribosomal RNA (rRNA) of *Ca*. Phycorickettsia was used together with unlabelled helper oligonucleotides. Transmission electron microscopy (TEM) was carried out using a standard protocol to study intracellular localization and ultrastructure of *Ca*. Phycorickettsia in vegetative cells of *T. minutus* CCALA 838 and *Pseudostaurastrum* sp. strain 10174. To avoid fixation artefacts, high-pressure freezing followed by freeze substitution fixation in OsO_4_ was applied.

**Table 1:** Eustigmatophyte strains tested for the presence of *Ca*. Phycorickettsia endosymbionts by PCR amplification and sequencing of 16S rDNA

Eustigmatophyte strain	PCR product	Identity of the sequence	GenBank accession number
*Characiopsis acuta* ACOI 1837	+	*Ca*. Phycorickettsia sp.	MH041630
*Characiopsis acuta* ACOI 456	+	*Ca*. Phycorickettsia sp.	MH041631
*Characiopsis saccata* SAG 15.97	−		
*Goniochloris sculpta* SAG 29.96	−		
*Monodus guttula* CCALA 828	+	*Microbacterium terricola*	not deposited
*Pseudostaurastrum enorme* SAG 11.85	−		
*Pseudostaurastrum limneticum* SAG 14.94	+	*Ca*. Phycorickettsia sp.	MH041632
*Pseudostaurastrum* sp. strain 10174	+	*Ca*. Phycorickettsia sp.	MH041633
*Pseudotetraëdriella kamillae* SAG 2056	−		
*Trachydiscus minutus* CCALA 838	+	*Ca*. Phycorickettsia trachydisci	identical to the 16S rRNA gene in the genome assembly (CP027845)
*Trachydiscus* sp. COBIEM 31	+	*Ca*. Phycorickettsia sp.	MH041634

The complete genome sequence of *Ca*. Phycorickettsia trachydisci (=*Phycorickettsia*) was assembled using 454 and Illumina reads obtained from DNA prepared from a culture of *T. minutus* CCALA 838 (see also [28]). Specifically, two scaffolds in a 454 reads-based assembly identified as coming from *Phycorickettsia* (based on sequence similarity, characteristic coverage, GC content) were manually connected using linking information from paired-end reads, and a continuous circular-mapping sequence was obtained by manual gap filling and polishing using 454 and Illumina reads. The final assembly was verified by visual inspection of 454 and Illumina reads mapped onto the genome sequence and further checking of apparent ambiguities. The final sequence was deposited at GenBank with the accession number CP027845. Protein-coding genes were annotated using the pipeline described before [30]. The genome was in parallel annotated using Prokka [31], discrepancies in structural gene annotation were identified and resolved by manual curation. Protein-coding genes of *Phycorickettsia* and other 17 members of Rickettsiales (Supplementary Table 2) were clustered into orthogroups using Orthofinder [32]. An analysis of metabolic pathways was aided by mapping the predicted *Phycorickettsia* proteome onto the KEGG database [33]. Moreover, BLAST searches [34] against the NCBI sequence databases (https://blast.ncbi.nlm.nih.gov/Blast.cgi) were used for more detailed evaluation of the identity and distribution of genes of special interest.

The bacterial 16S rDNA sequences were aligned using SINA (www.arb-silva.de/aligner/; [35]). Multiple alignments of protein sequences were built using MAFFT v7 (ref. [36]). The alignments were trimmed using GBLOCKS 0.91b (http://molevol.cmima.csic.es/castresana/Gblocks_server.html; [37]). A phylogenomic analysis of Rickettsiales was performed using a concatenation of trimmed protein sequence alignments of 116 genes exhibiting one-to-one orthology (as defined by Orthofinder). Maximum likelihood (ML) phylogenetic analyses of the 16S rDNA sequences were done using RAxML-HPC 8.2.10 (ref. [38]) at the CIPRES Portal (http://www.phylo.org/sub_sections/; [39]). ML phylogenetic analyses of protein sequence alignments were carried out using RAxML-HPC and IQ-TREE 1.5.5 (ref. [40]). The phylogenomic supermatrix was further analysed using Bayesian inference implemented in PhyloBayes MPI 1.7b [41] available on the MetaCentrum VO portal (metavo.metacentrum.cz). Details specific for individual phylogenetic analyses are provided in legends to respective figures.

## Results

### A new eustigmatophyte-associated lineage of Rickettsiaceae

**Fig. 1:**
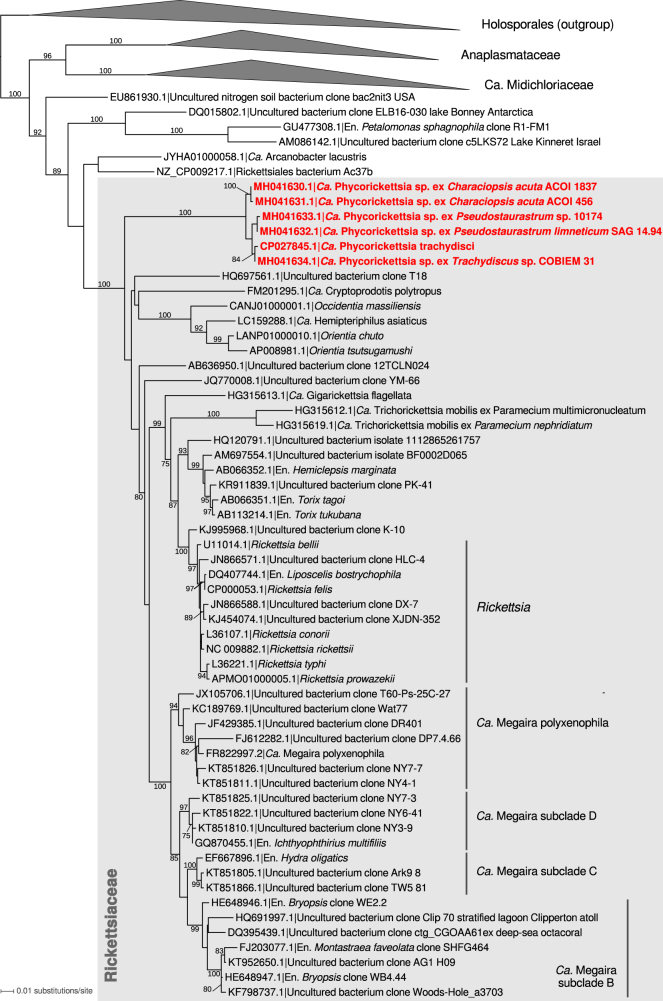
The phylogenetic position of *Candidatus* Phycorickettsia based on the 16S rDNA sequences. The maximum likelihood tree was inferred from an alignment of 1360 nucleotides using RAxML (GTR + Γ substitution model). Bootstrap values (calculated by the rapid bootstrapping algorithm of RAxML) are shown only when ≥ 75%

Inspection of an initial assembly of 454 reads obtained from total DNA isolated from a culture of *T. minutus* CCALA 838 revealed that some of the largest scaffolds exhibit apparent similarity to genes from Rickettsiaceae and are presumably derived from a bacterium representing a new lineage in the family based on a phylogenetic analysis of the 16S rRNA gene. We reasoned that this bacterium may be more generally associated with eustigmatophytes, so we designed PCR primers to specifically amplify the 16S rDNA of this bacterium or its close relatives, but not from other bacteria including other members of the Rickettsiaceae. Indeed, we could amplify products of the expected length (1110 bp) when using template DNA isolated from several distantly related eustigmatophytes (Table 1; Supplementary Figure 1). Except for the nonspecific product obtained from *Monodus guttula* CCALA 828 (matching the actinobacterium *Microbacterium terricola*), the remaining amplified sequences proved to be highly similar to the 16S rDNA sequence of the bacterium associated with *T. minutus*. The mutual differences of the eustigmatophyte-derived 16S rDNA sequences were at up to 10 out of 1035 positions compared, that is, <1%, whereas the most similar sequences from previously described bacterial species were up to 90% identical. A phylogenetic analysis confirmed that the Rickettsiaceae sequences obtained from the eustigmatophyte cultures constitute a tight separate clade deeply nested in the family Rickettsiaceae (Fig. 1).

We also carried out an *in silico* search for sequences with a specific affinity to the rickettsialean 16S rDNA associated with eustigmatophyte algae. We searched not only the non-redundant core GenBank database (including a vast number of sequences from environmental PCR-based 16S rDNA surveys), but also the NCBI database of metagenomic data, and ~2000 transcriptome assemblies from protists, algae and plants generated by MMETSP [42] and OneKP (https://sites.google.com/a/ualberta.ca/onekp/). The most similar full-length 16S rRNA sequences exhibited only up to 90% identity to the sequence from the *T. minutus*-associated bacterium and represented rickettsialean lineages not specifically related to the eustigmatophyte-associated bacterium. Notably, we likewise did not detect this bacterial lineage in the published genome assemblies of the genus *Nannochloropsis* (incl. *Microchloropsis*) or in our unpublished assemblies of DNA sequencing reads obtained from cultures of *Vischeria* sp. CAUP Q 202 and *Monodopsis* sp. MarTras21. However, we eventually identified two partial sequences from environmental DNA surveys (734 bp, KU191948.1; 878 bp, MF661830.1) that were 98–99% identical to the rickettsialean sequences we obtained from eustigmatophytes. The sequences are stated to originate from a microbial community associated with a moss and living in an aquaculture pond, respectively, but no further details of the origin of the sequences are available at the moment.

### *Candidatus* Phycorickettsia trachydisci: an endosymbiont of eustigmatophyte algae

**Fig. 2:**
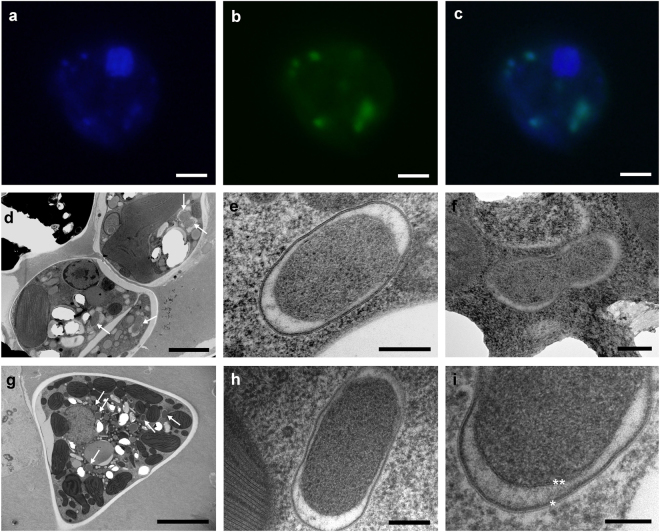
Bacterial endosymbionts in algal cells visualized by fluorescence and transmission electron microscopy (TEM). **a**-**c** Detection of *Candidatus* Phycorickettsia trachydisci by fluorescence *in situ* hybridization (FISH) in *Trachydiscus minutus* CCALA 838. **a** DAPI staining; (**b**) FISH with the probe 16S2 specific to 16S rRNA of *Ca*. Phycorickettsia trachydisci (for images obtained using the probe 16S1 see Supplementary Figure 2); (**c**) overlap of the two signals. **d**-**i** TEM of endosymbiotic bacteria in cells of *Trachydiscus minutus* CCALA 838 (**d-f**) and *Pseudostaurastrum* sp. strain 10174 (**g-i**). **d, g** Overall view of the representative cell ultrastructure, arrows indicate endosymbiotic bacteria in the cytoplasm. (**e**), (**h**) Longitudinal section of an endosymbiont. **f** A bacterium possibly dividing by transversal binary fission. **i** Detail showing the outer (asterisk) and the inner (double asterisk) bi-layer membranes with a less electron-dense periplasmic space. Bars 2 µm (**a**), (**b**), (**c**), (**d**), 200 nm (**e**), (**f**), (**h**), 5 µm **g** and 100 nm (**i**)

All known members of the order Rickettsiales are obligate intracellular bacteria [12]. To test whether the *T. minutus*-associated bacterium also occurs inside the algal cells rather than being an epibiont or a free-living co-inhabitant of the same culture, we employed FISH. Indeed, the fluorescence signal of two different probes specifically matching different regions of the 16S rRNA molecule of the novel bacterium appeared as distinct spots within cells of *T. minutus* CCALA 838 colocalizing with the signal of 4,6-diamidino-2-phenylindole (DAPI; Fig. 2a-c; Supplementary Figure 2a). Most, if not all, *T. minutus* cells carried one or more cells of the bacterial endosymbiont (Supplementary Figure 2b). Typically 5–10 endosymbiont cells were observed in a single *T. minutus* cell, although this may be an underestimation given the possibility of endosymbiont cells clumping, as well as some endosymbionts located outside of the focal plane. A more precise counting would require the use of confocal microscopy or serial section TEM.

TEM confirmed the presence of bacterial endosymbionts in *T. minutus* CCALA 838 (Fig. 2d-f), as well as in *Pseudostaurastrum* sp. strain 10174 (Fig. 2g-i). The bacteria were irregularly dispersed in the cytoplasm of the hosts (Fig. 2d, g). They were rod-shaped or oval, their dimensions varied and were not statistically different between strains –0.610 ( ± 0.086) × 0.391 ( ± 0.068) and 0.751 ( ± 0.198) × 0.392 ( ± 0.024) µm in *T*. *minutus* and *Pseudostaurastrum* sp. strain 10174, respectively. The ultrastructure of the endosymbionts was similar in both algal species. The bacterial protoplast generally appeared as an electron-dense material, surrounded by two bi-layer membranes presumably corresponding to the inner and the outer membrane of the conventional bacterial envelope (Fig. 2i). Between the membranes a less electron-dense material was observed, usually being larger at both poles of the endosymbionts (Fig. 2e, h). Division of the bacteria by transverse binary fission was occasionally observed (Fig. 2d, f).

Previously TEM was used to investigate the ultrastructure of another Rickettsiaceae endosymbiont of algae, specifically *Ca*. Megaira polyxenophila in cells of the green algae *Carteria cerasiformis* [15] and *Mesostigma viride* [16], with only the former study providing fine details of the endosymbiont morphology thanks to the application of high-pressure freezing and freeze substitution fixation. The traits reported here for the eustigmatophyte-inhabiting bacterium correspond well to the characteristics of *Ca*. Megaira polyxenophila in the two green algae, but some differences were noted. The eustigmatophyte endosymbionts are somewhat shorter and wider than *Ca*. Megaira polyxenophila [15, 16] and are not found preferentially at the cell periphery as the *C*. *cerasiformis* endosymbiont [15]. In addition, the bacteria in *T*. *minutus* and *Pseudostaurastrum* sp. strain 10174 do not exhibit an electron-lucent layer (considered to be a slime layer) surrounding cells of *Ca*. Megaira polyxenophila [15, 16]. The differences are consistent with the evidence from 16S rDNA sequences indicating that the eustigmatophyte endosymbiont is not specifically related to *Ca*. Megaira polyxenophila (Fig. 1).

The molecular data gathered for the eustigmatophyte endosymbiont allow us to conclude that it represents a novel genus-level lineage of the family Rickettsiaceae. In reference to the apparently exclusive association of the whole clade with algal hosts, we propose ‘*Candidatus* Phycorickettsia’, gen. nov., as a name of the clade, and ‘*Candidatus* Phycorickettsia trachydisci’, sp. nov., as the name for the endosymbiont occupying *T. minutus* CCALA 838. Whether the endosymbionts identified in the other eustigmatophytes should be classified as the same species needs to be resolved by further investigations.

### The uniqueness of *Phycorickettsia* confirmed by its genome sequence

To further illuminate the biology of *Ca*. Phycorickettsia trachydisci (*Phycorickettsia* for short), we assembled a complete genome sequence of the endosymbiont of *T. minutus* CCALA 838. The genome is circular mapping, 1 472 411 bp in length, with the average GC content of 34.07% and 1248 predicted protein-coding genes. All these numbers fit well into the range defined by other members of the order Rickettsiales (Table 2).

**Table 2:** General genomic features of *Ca*. Phycorickettsia trachydisci and other members of Rickettsiales

	Genome size (Mb)	% GC	Protein-coding genes
*Ca*. Phycorickettsia trachydisci	1.47	34.07	1 248
*Rickettsia* spp.	1.1–1.3	29	872–1 511
*Orientia* spp.	2.0–2.1	30	2 005–2 216
*Anaplasma* spp.	1.2–1.5	41–49	982–1 411
*Ehrlichia* spp.	1.2–1.5	27–30	961–1 158
*Wolbachia* spp.	1.1–1.5	34–35	900–1 423
Ca. Midichloria mitochondrii	1.18	36.6	1 245
*Ca*. Jidaibacter acnthamoeba	~2.4	34	2 267

**Fig. 3:**
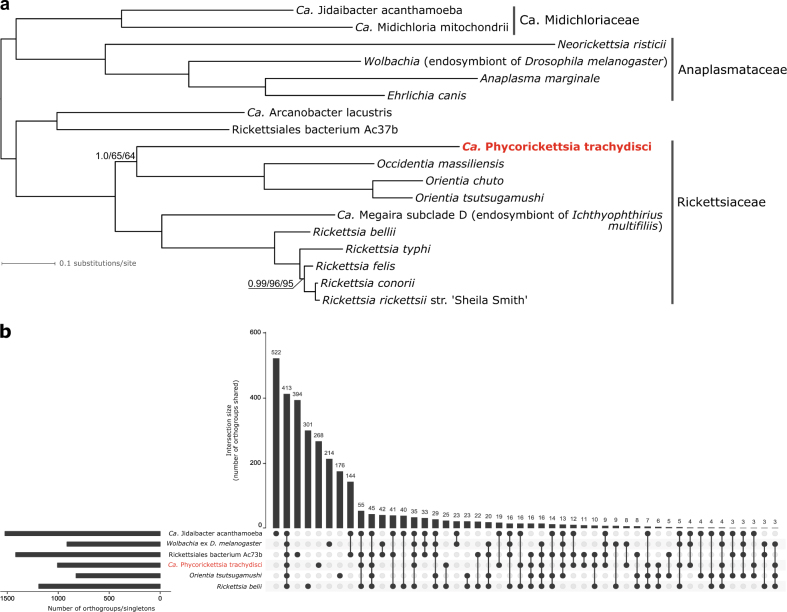
Phylogenomic and comparative genomic analysis of Rickettsiales including *Candidatus* Phycorickettsia trachydisci. **a** The phylogenetic position of *Ca*. Phycorickettsia trachydisci inferred from a supermatrix of 116 orthologous protein sequences (22 287 amino-acid positions). The tree displayed was inferred using PhyloBayes (CAT + GTR substitution model). In addition to posterior probabilities provided by PhyloBayes, branch support was also assessed by calculating nonparametric ML bootstrap values using RAxML and ultrafast ML bootstrap values using IQ-TREE (the LG4X + Γ model in both cases). Support values are shown only for branches that were not maximally supported in all three analyses. **b** Gene sharing among *Phycorickettsia* and five other representatives of Rickettsiales. Groups of orthologous genes (orthogroups) were defined using Orthofinder and a broader set of Rickettsiales members (the 18 species included in the tree in Fig. 3a). Patterns of orthogroup sharing represented by less than three orthogroups were omitted for simplicity. Orthogroups exclusive for a particular species correspond to a sum of species-specific clusters of paralogs and species-specific singletons. The plot was drawn using UpSetR [58]

To establish a basis for comparative analyses of the *Phycorickettsia* genome, we defined groups of putative orthologs (orthogroups) for protein-coding genes of this species and 17 other representatives of the order Rickettsiales. Among the 1831 orthogroups there were 116 represented by exactly one gene in each species; these were used to infer a phylogenetic tree of the order using the supermatrix-based approach (Fig. 3a). The resulting tree is consistent with both the analysis of the 16S rDNA (see Fig. 1) and previously published multigene analyses [43, 44], and confirms that *Phycorickettsia* constitutes a novel lineage of the family Rickettsiaceae. Specifically, the analyses suggest that *Phycorickettsia* is a deeply separated sister lineage of the clade comprising the genera *Orientia* and *Occidentia*, although this position is only weekly supported by maximum likelihood bootstrap values (yet receiving maximal support in the Bayesian analysis employing a presumably more realistic substitution model).

We then investigated the pattern of gene occurrence in Rickettsiales. Figure 3b plots sharing of orthogroups among *Phycorickettsia* and five selected species representing different branches of the order. *Phycorickettsia* clearly emerges as a distinctly different taxon, with the numbers of genes exclusive to the species, uniquely missing in the species, or shared in different combinations with the other five species comparable to the numbers exhibited by the other species included in the analysis. A similar view is provided by an analysis of orthogroup sharing based on comparing the *Phycorickettsia* genome and “pangenomes” of whole other lineages of Rickettsiales (Supplementary Figure 3). These results support the view of *Phycorickettsia* as a novel genus and indicate that it may display unique biological traits not seen in the Rickettsiales known so far.

### Notable aspects of the predicted metabolic and cellular functions of *Phycorickettsia*

**Fig. 4:**
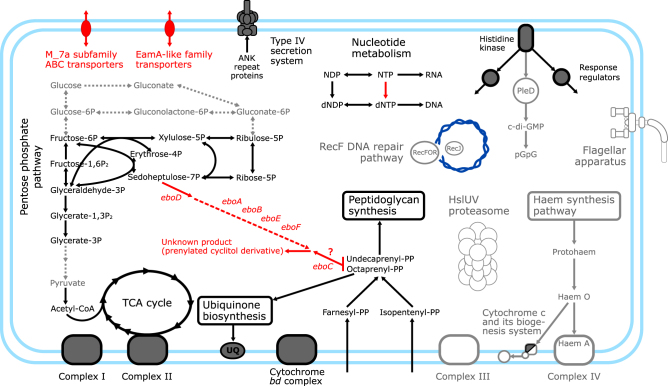
Notable biochemical and cellular features of *Candidatus* Phycorickettsia trachydisci gleaned from its genome sequence. The scheme shows selected metabolic pathways and molecular components of the *Phycorickettsia* cells (those that are specifically discussed in the text). Reactions (arrows) and molecular components present in *Phycorickettsia* and common in other Rickettsiales are shown in black, those absent from *Phycorickettsia* yet common in other Rickettsiales are in grey, and those unique for *Phycorickettsia* are in red. Biochemical steps generally missing from Rickettsiaceae (including *Phycorickettsia*) are shown as dotted grey arrows. Supplementary Table 3 provides sequence IDs of the proteins underpinning the pathways and modules in the figure (colour figure online)

To obtain specific functional predictions from the *Phycorickettsia* genome, we analysed its gene content by mapping the genes onto functional pathways defined in the KEGG database and by performing targeted evaluation of genes of special interest, such as those exhibiting an interesting phyletic pattern. Here we focus on the most salient results of these analyses. A graphical summary is provided in Fig. 4, the genes concerned are listed in Supplementary Table 3.

Like other Rickettsiales studied so far [43, 45, 46], *Phycorickettsia* exhibits components of the type IV secretion system (T4SS). The role of this system presumably is to deliver effector proteins from the endosymbiont to host cells. One of the known classes of rickettsialean effector proteins is characterized by the presence of ankyrin (ANK) repeats. It is, therefore, interesting to note that the *Phycorickettsia* genome codes for a highly expanded set of ANK repeat proteins. We specifically identified 142 such proteins, which is the highest number recorded so far for the whole Rickettsiales order (Supplementary Table 3). However, whether all these proteins are delivered to the host and why *Phycorickettsia* potentially deploys such a broad battery of effectors remains unknown.

The core carbon metabolism is similarly simplified in *Phycorickettsia* as in other Rickettsiales. There are no enzymes present that would enable metabolizing glucose, and the glycolysis pathway is incomplete due to the absence of enzymes converting glycerate-3P to pyruvate. However, unlike *Orientia tsutsugamushi* [47], *Phycorickettsia* has retained the pyruvate dehydrogenase complex and a complete tricarboxylic acid (TCA) cycle. An interesting aspect of the carbon metabolism in *Phycorickettsia* is the presence of all enzymes of the non-oxidative part of the pentose phosphate pathway. The pathway was considered to be missing in Rickettsiaceae, until a recent genomic study identified it in the endosymbiont of the ciliate *Ichthyophthirius multifiliis* (*Ca*. Megaira subclade D) and in a member of the “Torix group” of *Rickettsia* [46]. The functional significance of the differential presence of the pathway in Rickettsiaceae is, however, unclear.

Important insights into the *Phycorickettsia* biology were gleaned from investigating genes and pathways that are notably absent due to lineage-specific loss (Supplementary Table 3). Particularly interesting is a substantial reduction of the respiration chain of the bacterium. It lacks not only the complex IV, independently lost also in *O. tsutsugamushi* strain Ikeda [47] and some other Rickettsiales, but also the complex III (additionally not found only in the incomplete genome sequence of *Ca*. Arcanobacter lacustris among all other Rickettsiales analysed). Truly unique for *Phycorickettsia* is the absence of cytochrome *c* and some of the components of the machinery involved in cytochrome *c* biogenesis. Reduced ubiquinone generated by the complexes I and II is in *Phycorickettsia* most likely re-oxidized by the alternative terminal oxidase cytochrome *bd* [48], which is present also in some other Rickettsiales in parallel to complexes III/IV. Strikingly, *Phycorickettsia* has completely dispensed with the haem biosynthesis pathway that is otherwise at least partially present in all other lineages of Rickettsiales investigated. This may reflect a decreased need for the haem due to the loss of complexes III and IV and cytochrome *c*. However, the presence of Complex II (succinate dehydrogenase) and the cytochrome *bd* complex, both of which also include haem prosthetic groups [48, 49], indicates that *Phycorickettsia* imports haem from its host.

Simplification specific for *Phycorickettsia* goes beyond metabolic pathways (Supplementary Table 3), as documented by the unique absence of genes for all proteins involved in the RecF recombination pathway of double strand breaks repair (see [50]). Also missing are genes for both subunits of the HslUV (or HslVU) complex, a common bacterial AAA+ proteases involved in the turnover of cellular proteins [51]. Unusual among Rickettsiales is also the absence of a response regulator with an effector domain corresponding to diguanylate cyclase (synthesizing cyclic di-GMP) and of an EAL-type c-di-GMP phosphodiesterase (see [52]). We did not detect any other proteins predicted to produce or degrade c-di-GMP, indicating that in contrast to other Rickettsiales, *Phycorickettsia* has completely dispensed with the c-di-GMP-based signalling. On the other hand, the absence of genes for the flagellum in the *Phycorickettsia* genome is a trait shared with many members of Rickettsiales, including most species of Rickettsiaceae sequenced so far (Supplementary Table 3).

*Phycorickettsia* includes 245 genes without orthologs in other 17 Rickettsiales included in the analysis. In all, 179 of them are true orphans (defined as genes with no BLASTp hits in other organisms with E-value < 1.10e–4), the rest (66) are candidate acquisitions by horizontal gene transfer (HGT) specific for the *Phycorickettsia* lineage. Some of the latter genes are noteworthy for their functional annotation (Supplementary Table 3). Two of them represent the *nrd*DG operon that encodes anaerobic (i.e., oxygen sensitive) ribonucleoside-triphosphate reductase (and its activase) catalysing production of dNTPs for DNA synthesis [53]. The presence of this enzyme in *Phycorickettsia* suggests that the bacterium experiences prolonged anaerobic conditions, although this might seem surprising for an endosymbiont of an oxygenic phototroph. Exclusive for *Phycorickettsia* is also the presence of genes encoding ATP-binding cassette (ABC) transporters of the M_7a subfamily (three paralogs) and genes representing the EamA-like transporter family (two paralogs; Supplementary Table 3). These transporters point to unique aspects of the metabolic interaction between *Phycorickettsia* and its eukaryotic host, but their actual substrate specificity cannot be readily predicted from the sequence data only.

### The unexpected presence of the *ebo* operon in *Phycorickettsia*

**Fig. 5:**
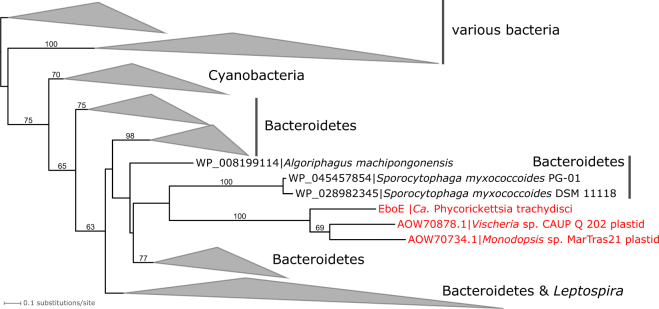
Phylogenetic analysis of the EboE protein demonstrating the specific relationship of the *ebo* operon from *Ca*. Phycorickettsia trachydisci and the plastid genomes of *Vischeria* sp. CAUP Q 202 and *Monodopsis* sp. MarTras21. The tree was inferred (based on a multiple alignment comprising 314 amino-acid positions) using RAxML (LG4X + Γ substitution model). Bootstrap values (from rapid bootstrapping) are shown only when > 50%. For simplicity, clades comprising solely bacterial sequences were collapsed as triangles without showing individual constituent sequences. See also Supplementary Figure 5

The most intriguing finding that emerged from the comparative analysis of the *Phycorickettsia* genome was the finding of the *ebo* operon (Supplementary Table 3). As explained in Introduction, we recently described this operon when investigating the plastid genomes of the eustigmatophytes *Vischeria* sp. CAUP Q 202 and *Monodopsis* sp. MarTras21 [29]. The *ebo* operon in *Phycorickettsia* has the same architecture as the one in the two plastid genomes and in some bacterial groups, particularly Bacteroidetes and Cyanobacteria (Supplementary Figure 4), that is, is of the “ABCDEF type” (the letters reflect the order of the six *ebo* genes; see [29]). The identification of the *ebo* operon in *Phycorickettsia* is indeed noteworthy, as we detected neither the operon as a whole nor homologues of individual *ebo* genes in any other member of Rickettsiales with the genome sequence available. Strikingly, in phylogenetic analyses each of the six Ebo protein sequences from *Phycorickettsia* constituted a robustly supported clade with its homologues encoded by the two eustigmatophyte plastid genomes (Fig. 5; Supplementary Figure 5).

We previously proposed that the substrate for the enzyme encoded by the *eboD* gene, representing a novel subgroup of the sugar phosphate cyclase superfamily, could be sedoheptulose-7-phosphate [29]. Interestingly, this substance is predicted to occur in *Phycorickettsia* owing to the presence of the pentose phosphate pathway (see above; Fig. 4), so our hypothesis about the substrate specificity of the EboD protein is consistent with the predicted metabolic capacity of *Phycorickettsia*. Another *ebo* gene (*eboC*) codes for a novel member of the UbiA superfamily of polyprenyl transferases with an unknown substrate specificity [29]. Our analysis of the *Phycorickettsia* gene content suggests that the bacterium relies on externally supplied isopentenyl-pyrophosphate and farnesyl-pyrophosphate to synthesize two polyprenyl forms—octaprenyl-pyrophosphate and undecaprenyl-pyrophosphate (Fig. 4). The former is involved in ubiquinone synthesis and the latter serves in peptidoglycan synthesis as a carrier of saccharide groups. We hypothesize that one of these polyprenyls is also the substrate for EboC in *Phycorickettsia* and possibly other species, given their wide occurrence in bacteria. The functional prediction for the remaining Ebo proteins is less clear [29], so at present we cannot link them to specific reactions in *Phycorickettsia* (Fig. 4).

## Discussion

Bacterial endosymbionts are being increasingly recognized as an important ingredient of algal life [54]. However, eustigmatophytes have so far not been included in the list of algal taxa known to harbour intracellular bacteria, so our work unveils a new dimension of the biology of this taxon. The fact that the eustigmatophyte endosymbiont represents a new genus-level lineage of Rickettsiaceae is also notable, as the previously documented members of this family found in protistan hosts all belong to the phylogenetically unrelated genus *Ca*. Megaira (Figs. 1, 3a).

The relatively large and gene-rich genome (Table 2) and not particularly rapid sequence evolution of *Phycorickettsia* genes (Figs. 1, 3a) suggest that this bacterium is not an obligate endosymbiont transmitted purely by vertical inheritance (for a review of genomic signatures of obligate endosymbionts see, e.g., [55]). On the other hand, the phylogenetic relationships within the *Phycorickettsia* clade (Fig. 1) reflect the phylogenetic relationships of the algal hosts (Supplementary Figure 1). Specifically, 16S rDNA sequences obtained from representatives of the clade Goniochloridales (two *Trachydiscus* spp. and two *Pseudostaurastrum* spp.) are monophyletic to the exclusion of the sequences obtained from the representative of the order Eustigmatales, *Characiopsis acuta*. This would be consistent with a history of co-evolution of the bacteria and the host algae starting before the split of the two main eustigmatophyte lineages. However, the low degree of 16S rDNA sequence divergence between the different *Phycorickettsia* lineages (<1% of nucleotide differences) speaks against this interpretation. 18S rDNA sequences of eustigmatophyte species infected by *Phycorickettsia* differ in up to ~10% nucleotide positions and molecular clock-based analyses of the ochrophyte phylogeny suggested that eustigmatophyte constituting the order Eustigmatales diverged around 120 MYA [56]. Members of Goniochloridales were not included in that analysis, but the divergence between Goniochloridales and Eustigmatales would obviously be estimated as an even older event. It is unlikely that bacterial lineages would differentiate in their 16S rDNA so little after such a long time of separate evolution.

We thus propose that *Phycorickettsia*, like other members of Rickettsiales [12], can be transmitted from one host to another, although the mechanism of the transmission remains unclear. Eustigmatophyte vegetative cells are covered by the cell wall [19], so we speculate that naked zoospores might be the actual life stage providing a route for *Phycorickettsia* to invade a new eustigmatophyte host. This notion is consistent with the apparent absence of *Phycorickettsia* from the genomic and transcriptomic data from eustigmatophytes unknown to produce zoospores, including multiple *Nannochloropsis* (and *Microchloropsis*) species and *Monodopsis* sp. MarTras21 (see [19]). At the moment, *Vischeria* sp. CAUP Q 202 is the only zoosporic eustigmatophyte that clearly lacks the *Phycorickettsia* endosymbiont (evidenced by our genome and transcriptome sequencing), whereas the negative results of PCR experiments for some other eustigmatophytes (Table 1) should not be considered as definite proof of the *Phycorickettsia* absence from the given algal strains. We are presently further surveying the eustigmatophyte diversity by genome and/or transcriptome sequencing to map the *Phycorickettsia* occurrence and to illuminate the nature of its association with algal hosts.

It is also important to further check the possible *Phycorickettsia* host range beyond eustigmatophytes. We did not detect *Phycorickettsia* 16S rDNA/rRNA sequences in genomic and transcriptomic data from non-eustigmatophyte organisms, including hundreds of diverse heterotrophic and photosynthetic protists of all main groups. It is noteworthy that the *Phycorickettsia* 16S rRNA is present in the transcriptome assembly we obtained by sequencing polyA-selected RNA isolated from *T. minutus* (data not shown), confirming that the bacterium would be recorded in transcriptomic projects from other organisms if present. However, this does not necessarily imply strict specificity of *Phycorickettsia* to eustigmatophytes. Indeed, 16S rDNA/rRNA sequences from *Ca*. Megaira seem to be similarly scarce in the organism-derived sequence resources, despite the fact that this lineage has been reported from a number of different hosts. However, it is frequently encountered in environmental DNA samples (Fig. 1), contrasting thus with the near lack of *Phycorickettsia* in environmental surveys. Differences in the host type and abundance may account for the different representation of the two rickettsiacean lineages.

Similar to other Rickettsiaceae, the metabolic capacity of *Phycorickettsia* from *T. minutus* predicted by its genome sequence is limited, indicating that the bacterium relies on its host not only for the source of energy, but also for the source of many substances for catabolism, for example, haem and isoprenoid precursors (Fig. 4), purines, pyrimidines, various amino acids and enzyme cofactors (data not shown). However, it is premature to conclude that *Phycorickettsia* is a pure parasite. The discovery of the *ebo* operon in the *Phycorickettsia* genome raises an intriguing possibility that *Phycorickettsia* can synthesize a substance that is beneficial for its host. Although we do not know what the actual product of the Ebo proteins is, the very fact that the operon occurs in plastid genomes of two distantly related eustigmatophytes (both apparently uninfected by *Phycorickettsia*) suggests that its activity is useful for the algae. Elucidating the actual biochemical role of the *ebo* operon is thus critical for better understanding of the physiology of the *Phycorickettsia*–eustigmatophyte interaction.

We previously concluded that the most likely bacterial donor of the eustigmatophyte *ebo* was a bacterium from the phylum Bacteroidetes [29]. The close relationship of *ebo* genes from *Phycorickettsia* to those from the eustigmatophyte plastid genomes, together with the absence of the *ebo* operon from Rickettsiales other than *Phycorickettsia*, indicate that the *ebo* operon in *Phycorickettsia* ultimately originated from the same source, that is, a bacterium of the phylum Bacteroidetes, as the *ebo* operon in eustigmatophyte plastid genomes. Furthermore, our phylogenetic analyses consistently suggest that the *ebo* operons in *Vischeria* and *Monodopsis* are monophyletic to the exclusion of the operon in the *Phycorickettsia* genome (Fig. 5; Supplementary Figure 5). This indicates a genetic exchange event that occurred between the *Phycorickettsia* lineage and eustigmatophytes before the divergence of *Vischeria* and *Monodopsis* (but possibly only after the Goniochloridales-Eustigmatales split, as the plastid genome of *T. minutus* lacks the *ebo* operon; [29]). However, the phylogenetic analysis itself does not tell the direction of the transfer. Investigating the genomic neighbourhood of the *ebo* operon in the eustigmatophyte plastid genomes and in *Phycorickettsia* does not provide any further hints either. In eustigmatophytes the operon is inserted neatly between conserved and syntenic plastid genes [29]. In *Phycorickettsia*, the operon is flanked on the 5’-end by three genes without discernible homologues elsewhere and on the 3’-end by a putative hydrolase-encoding gene with closest homologues in various non-rickettsialean bacteria and eukaryotes, hence also putatively gained by HGT into the *Phycorickettsia* lineage, but certainly not from a plastid.

Although broader genomic sampling of both eustigmatophytes and *Phycorickettsia* relatives are required to resolve the direction of the *ebo* operon transfer between these two lineages, the general prevalence of bacteria-to-eukaryotes gene flow (e.g., [57]) leads us to favour the hypothesis that the eustigmatophyte plastid lineage was the recipient rather than the donor. This would imply that the *ebo* operon was first acquired by an ancestor of *Phycorickettsia* from another bacterium, most likely a member of Bacteroidetes (see [29]). Furthermore, we speculate that this acquisition facilitated the establishment of *Phycorickettsia* as eustigmatophyte endosymbiont due to the benefits conferred by the *ebo* operon activity to the algal host. An early representative of this bacterial lineage infecting an ancestor of *Vischeria* and *Monodopsis* passed the *ebo* operon to the plastid genome of the host cell. Thus, we interpret the presence of the *ebo* operon in the *Vischeria* and *Monodopsis* plastid genomes as an evolutionary footprint of a past presence of a *Phycorickettsia*-related endosymbiont, implying that *Phycorickettsia* is a long-term evolutionary partner of eustigmatophytes. Future exploration of genomes of additional *Phycorickettsia* and eustigmatophyte lineages will enable us to test this hypothesis.

## Electronic supplementary material

Supplementary Materials and Methods

Supplementary figures

Supplementary Tables 1 & 2

Supplementary Table 3
