# Supplementary material for: A gene transfer event suggests a long-term partnership between eustigmatophyte algae and a novel lineage of endosymbiotic bacteria
Source: ISME J. 2018 Jun 7;12(9):2163–75. doi: 10.1038/s41396-018-0177-y (PMC6092422; doi:10.1038/s41396-018-0177-y)
Supplement: Supplementary file 1 — Supplementary Materials and Methods [file 41396_2018_177_MOESM1_ESM.pdf]

Supplementary Materials and Methods

Algal strains

Trachydiscus minutus CCALA 838 is a strain reported by us previously (Přibyl *et al.*, 2012) and continuously maintained in our lab in the BBM medium (Nichols, 1973) or on agar plates (2% agar in Zehnder medium; Staub, 1961) at the light intensity of 40 µmol m-2 s-1 PhAR and temperature 24°C. *Pseudotetraëdriella kamillae* SAG 2056, *Goniochloris sculpta* SAG 29.96, *Pseudostaurastrum limneticum* SAG 14.94 and *Pseudostaurastrum enorme* SAG 11.85 were ordered from the SAG culture collection (http://www.uni-goettingen.de/en/www.uni-goettingen.de/de/184982.html) and grown as *T. minutus*. Two strains of *Characiopsis acuta*, ACOI 456 and ACOI 1837, were obtained from the ACOI culture collection (http://acoi.ci.uc.pt/) and grown in liquid Desmidiacean medium (Schlösser, 1994) with pH of 6.4 to 6.6, at 20°C, a 12:12 h photoperiod and under 10 µmol/m^2/s light intensity provided by cool white fluorescent lamps. *Pseudostaurastrum* sp. strain 10174 was isolated from the plankton of a pond in the town České Budějovice, Czech Republic (48.9719275N, 14.4575853E) in November 2009. A clonal, non-axenic culture was derived from a single cell isolation and has been maintained in liquid Zehnder medium (Staub, 1961), at a light intensity of 20 µmol m^{-2} s^{-1} and room temperature. *Trachydiscus* sp. strain COBIEM 31 was isolated from the plankton of a fishpond near the town Hluboká nad Vltavou, Czech Republic (49.0425475N, 14.4270672E) in April 2010. A clonal culture was isolated and purified by single cell isolation and agar plate method. An axenic culture has been maintained on agar slants (2% in Zehnder medium) at a light intensity of 20 µmol m^{-2} s^{-1} and at a temperature of 8°C. The eustigmatophyte identity of the newly isolated strains and the two ACOI strains so far lacking published molecular taxonomic characterization was confirmed by sequencing their 18S rRNA genes (see below).

Amplification and sequencing of the 16S rRNA and 18S rRNA genes

For newly isolated strains or strains newly acquired from culture collections, DNA was isolated from liquid or agar cultures using Plant DNA isolation kit (Invitek, Germany) following manufacturer's recommendation and diluted ten times. For PCR experiments we additionally used DNA samples from *Characiopsis saccata* SAG 15.97, *Monodus guttula* CCALA 828, and *T. minutus* CCALA 838 whose preparation was reported before (Přibyl *et al.*, 2012; Fawley *et al.*, 2014). The 18S rDNA regions of the newly characterized algal strains (*Trachydiscus* sp. COBIEM 31, *Pseudostaurastrum* sp. strain 10174) were amplified and sequenced using primers described in Katana *et al*. (2001). Primers for amplification and sequencing of 16S rDNA of the rickettsialean endosymbiont of eustigmatophytes (PhycorickettsiaF2: 5'-TAGTACGGAATAACCCTTGG-3'; PhycorickettsiaR2: 5'-ATGGATTTGCTCCACCTTAC-3') were designed based on the sequence identified in the genome assembly of the endosymbiont of *T. minutus* CCALA 838. The target genes were amplified in 20 µl PCR reaction mix containing 0,5U MyRED Taq polymerase (Bioline, United Kingdom), 1x MyTaq reaction buffer, 8 µmol of each primer, PCR grade water, and 1 µl of the DNA template. Amplification was set up as follows: 95°C for 3 minutes, 35 cycles of 95°C for 30 seconds, 52°C for 30 seconds, 72°C for 90 seconds, followed by final extension 72°C for 10 minutes and constant incubation at 10°C. Amplification of the 18S rRNA gene using standard forward and reverse primers (as above) was used as a positive control for each DNA sample. PCR products representing a putative 16S rDNA region were separated by electrophoresis (5V/cm) in 1% agarose gel and then purified using Gel/PCR DNA Fragments Extraction Kit (Geneaid, Taiwan) when a positive result was

obtained. PCR products were sequenced using the Sanger dideoxy method by the sequencing company Macrogen (Korea) or by the sequencing service of the Faculty of Science, Charles University in Prague. Sequencing reads were assembled using the Sequencher software (Gene Codes Corporation, USA). The newly determined 16S and 18S rRNA gene sequences were deposited at GenBank with accession numbers MH041630-MH041634 and MH045495-MH045496.

Fluorescence in situ *hybridization*

Two oligonucleotide probes specifically targeting 16S rRNA of the rickettsialean endosymbiont of *T. minutus* CCALA 838 (16S1: 5'-AATCTCCCCGACATCTAACAT-3'; 16S2: 5'-GCAGAATGTTTAACGCGTTAG-3') were designed by inspecting a broader alignment of 16S rDNA sequences so that the probe contained at least three mismatches to the non-target bacteria. Horseradish peroxidase (HRP)-conjugated probes were purchased from biomers.net (Serco Industrial Park West, Ulm/Donau, Germany). Four unlabelled helper oligonucleotides (AAT_Helper_1: 5’ GTTAGCTACGAAACCGAGGA-3’, AAT_Helper_2: 5’-TCATCGTTTACAGCGTGGAC-3’, GCA_Helper_1: 5'-CTAGGAAACCGAGGAGTAATCT-3' and GCA_Helper_2: 5'-AATCTTGCGATCGTACTCCC-3') were used to improve the accessibility of the target region of the 16S rRNA molecule (Fuchs *et al.*, 2000). About 7.5 mL of a dense culture of *T. minutus* CCALA 838 grown in AF-6 media was fixed in 2.6% formaldehyde (final concentration) for 30 min on ice, incubated with 1% TritonX-100 at room temperature for 30 minutes, rinsed with PBS and then collected onto a 47-mm-diameter 0.8-µm-pore polycarbonate filter (Sterlitech, Kent, WA, USA). The filter was dehydrated in a graded methanol series (50%, 80%, and 100% for 5 min each). After the filter was air-dried, it was cut into eight pieces using a clean blade. Inactivation of endogenous peroxidase activity, hybridization in 25% formamide, signal amplification, and DAPI counterstaining procedures were followed as described by Kim *et al*. (2011). Microscopy was done within two days of staining using an Olympus DP73 camera.

Transmission electron microscopy (TEM)

Algal cell suspensions were concentrated by centrifugation, the compact pellet was mixed with a drop of 20% bovine serum albumin (BSA) and centrifuged for 10 minutes (3 000 rpm). Small drop (~1 µl) of cells was directly transferred into a flat specimen carriers (FSC) made of gold-plated copper (height 0.5mm, inner diameter 1.2mm, depth 0.2mm, Leica) mounted in a rapid loader for high pressure freezing (HPF). With the help of a loading station, the rapid loader with FSC were gently pushed into a loading device pod placed in rapid transfer system (RTS) of HPF. The specimen carrier was tightly sealed by pressing it against a surface made of black diamond. RTS moved the specimen into a high pressure freezer (HPF) EM PACT2 (Leica Microsystems, Vienna, Austria) where the samples were frozen at a pressure of about 2005 bar in five seconds. Following freezing, the FSC were separated from the loading device pods under liquid nitrogen and transferred into screw-capped containers filled with the frozen substitution medium (2% OsO_4 in 100% acetone) pre-cooled to -90 °C. The containers were placed to a freeze-substitution system Leica AFS (Leica Microsystems). The substitution was let proceed for several days with a protocol starting at -90 °C for 96 hours, followed by warming at 5 °C/h to -20 °C. After 24h at -20 °C, warming was continued at 3 °C/h to 3 °C. This was followed by 8 h at 3 °C, and 18h at 4 °C. Subsequently, the specimens were rinsed three times with anhydrous acetone and infiltrated in a mixture of acetone and Spurr resin (changing the ratio in three steps, each taking 1 h at the room temperature, from 2:1 via 1:1 to 1:2).

After overnight incubation in pure Spurr, the material was released from the HPF carriers, and whole specimens carriers were embedded in fresh resin let polymerize at 62 °C for 48 h. Ultrathin sections (70 nm) were cut using an ultramicrotome (UCT, Leica) and collected on copper grids. The grids were contrasted in ethanolic uranyl acetate and lead citrate, and observed in a JEOL 1010 TEM (Jeol, Japan) at an accelerating voltage of 80 kV. Images were recorded with a Mega View III camera (SIS GmbH, Germany) and processed using Adobe Photoshop 7.0 (Adobe Systems, USA).

Sequencing, assembly, and annotation of the Candidatus *Phycorickettsia trachydisci genome*
The genome of *Candidatus* Phycorickettsia trachydisci was sequenced using 454 and Illumina reads obtained from DNA prepared from a culture of *T. minutus* CCALA 838 (see Ševčíková *et al.*, 2015). The 454 GS FLX sequencer was used to obtain the following sequencing data: (1) over 460 000 shotgun reads from four sequencing runs (with the average read length from ~200 to ~500 bp, depending on the run); (2) over 3 200 000 reads from a 3-kb paired-end library, resulting in ~1 110 000 single (unpaired) reads and 2 150 000 paired reads (with the average read length of ~170 to 340 bp, depending on the run). Over 15 800 000 Illumina pair-end reads (101 bp) were generated by GATC, Konstanz, Germany. The 454 reads were assembled using Newbler 2.6 (Roche), resulting in 7 191 scaffolds and ~70 Mb bp. Two scaffolds were identified as representing the genome of a novel Rickettsiaceae bacterium based on sequence similarity, characteristic coverage, GC content, and linking information from paired-end reads. The scaffolds were assembled into a continuous circular-mapping sequence by iterative extending, gap filling and polishing using both 454 and Illumina reads. The assembly procedure and validation of the final assembly was aided by mapping the reads onto assembly intermediates or the complete genome sequence using Bowtie 2.1.0 (Langmead and Salzberg, 2012) and the CLC Genomics Workbench 11.0 and visual inspection of the alignments in GenomeView 2020 (Abeel *et al.*, 2012) and Tablet 1.14.04.10 (Milne *et al.*,2013). Conversion of the output of Bowtie in the SAM format into the BAM format readable by GenomeView was done using SAMtools 0.1.19 (Li *et al.*, 2009). The latter programme was also used to extract paired reads conflicting with the pre-final assembly. These were manually evaluated, leading to the identification of one misassembled region that was then fixed to remove the conflict with the paired reads. The average coverage of the final assembled sequence was 55.44 by 454 reads and 62.87 in Illumina reads.

Ribosomal RNA and tRNA genes were identified using RNAmmer (Lagesen *et al*., 2007) and tRNAscanSE (Lowe and Eddy, 1997), respectively. Coding sequence (CDS) prediction was achieved using the programmes AMIGene (Bocs *et al.*, 2003) and Prodigal (Hyat *et al.*, 2010). The Prokka software (Seemann, 2014) with RNAmmer as an rRNA predictor was used to obtain an independent annotation of the genome. The annotated features were visualised and manually curated in Artemis (Rutherford *et al*., 2007). Gene sets predicted by the two separate procedures were compared and all discrepancies (genes present in only one set, different prediction of the initiation codon etc.) were manually checked and resolved based on comparison to homologous sequences. Predicted intergenic regions were used as queries for BLASTx searches (Altschul *et al.*, 1997) to detect possible short protein-coding genes missed by both methods. One short gene (*secE*) was identified and integrated into the annotation based on its suspicious absence revealed by comparative genomic analyses. Functional gene annotation was primarily obtained using the PipRick pipeline (El Karkouri *et al.*, 2017), except for the genes annotated only by Prokka and genes of special interest (listed in Supplementary Table 3) annotated based on targeted analyses described in the following paragraph.

Comparative genomic analyses

Protein-coding genes of *Ca.* Phycorickettsia trachydisci and a set of 17 other selected members of the order Rickettsiales (Supplementary Table 2) were clustered into orthogroups using Orthofinder (Emms and Kelly, 2015) with default settings. The output of this programme was processed using in-house Python scripts to obtain counts of orthogroups and singletons in different subsets of the species analysed. The presence or absence of various metabolic pathways and functional modules in *Phycorickettsia* was evaluated by mapping the predicted protein set onto the Kyoto Encyclopedia of Genes and Genomes (KEGG) database (http://www.genome.jp/kegg/; Kanehisa *et al.*, 2016). Genes (proteins) of special interest were further analysed using BLASTp searches against the nr protein sequence database at NCBI or the predicted proteins sequences from *Phycorickettsia*. Notable gene absences in the *Phycorickettsia* genome were checked by BLASTp and tBLASTn searches. Two enzymes of the haem biosynthesis pathway indicated to be absent by querying the KEGG database were identified as follows. Firstly, it turned out that Rickettsiales exhibit the HemJ form of protoporphyrinogen IX oxidase (see Kato *et al.*, 2010) not yet integrated in KEGG. Secondly, In Rickettsiaceae we identified a divergent homolog of HemD absent from other Rickettsiales (which exhibit a conventional HemD form) that we hypothesize is the actual uroporphyrinogen-III synthase in this lineage. The genes of special interest (dealt specifically with in the main text) are included in Supplementary Table 3 as the respective orthogroups defined by Orthofinder (see) above, except for HemD, where the two orthogroups (Rickettsiaceae divergent HemD homologs and non-Rickettsiaceae conventional HemD) were combined. Absence from *Phycorickettsia* of proteins domains involved in the c-di-GMP metabolism (GGDEF and EAL) was checked by searching the predicted proteome of the species with HMMER 3.1b1 (Eddy, 2009) and using the respective profile HMMs (PF00990 and PF00563) obtained from the Pfam database (http://pfam.xfam.org/; Finn *et al.*, 2016). Ankyrin (ANK) repeat proteins in *Phycorickettsia* and the 17 other rickettsialean genomes analysed were identified by searching the respective predicted protein sets with SMART (http://smart.embl-heidelberg.de/; Letunic *et al.*, 2015). The search was done with the option of identification of PFAM domains set on. Putative ANK repeat proteins were then identified as those including at least one significant match against any ANK repeat variant, i.e. the ANK profile as defined by SMART and the PFAM domains belonging to the clan *Ank* (http://pfam.xfam.org/clan/CL0465). The subfamily assignment of the ABC transporters that *Phycorickettsia* harbours uniquely among Rickettsiales is based on using the ABCdb database (Fichant *et al.*, 2006).

Building and processing multiple sequence alignments

Newly determined eustigmatophyte 18S rDNA sequences (Supplementary Table 1) and relevant newly released sequences (*Tetraëdriella subglobosa* - KX373531.1; *Trachydiscus* sp. LCR-Awa-9-2 - KM014497.1) were manually added to the multiple alignment employed in our previous study (Ševčíková *et al.*, 2016). A set of 16S rDNA sequences for phylogenetic analyses was assembled by combining the newly determined sequences from *Ca.* Phycorickettsia with available sequences representing different lineages of Rickettsiales (in-group) and Holosporales (out-group) or identified as most similar to the sequences from *Ca.* Phycorickettsia by BLASTn searches against the nr nucleotide database at NCBI (https://blast.ncbi.nlm.nih.gov/Blast.cgi). The selection of the sequences retained in the final analysis was based on a preliminary tree inferred from a larger dataset and considering previously published phylogenetic analyses of Rickettsiales. The 16S rDNA sequences were

aligned using SINA (www.arb-silva.de/aligner/; Pruesse *et al.*, 2012). Multiple alignments of protein sequences were built using MAFFT v7 (Katoh and Standley, 2013), either *de novo* (proteins from Rickettsiales employed in the phylogenomic analysis, see below) or using the "--add" function of MAFFT (to add sequences of the six Ebo proteins to multiple alignments of their respective homologs employed in our previous study – Yurchenko *et al.*, 2016). The alignments were processed by removing unreliably aligned or too divergent regions using the GBLOCKS 0.91b tool (http://molevol.cmima.csic.es/castresana/Gblocks_server.html; Castresana, 2000) with the settings keeping the maximal number of positions in the final alignment. For a phylogenomic analysis of Rickettsiales, trimmed protein sequence alignments of 116 genes exhibiting the one-to-one orthology (as defined by Orthofinder, see above) were concatenated using FASconCAT (Kück and Meusemann, 2010). Sequence alignments used in the study are available from the corresponding author upon request.

Tree inference and processing

Maximum likelihood (ML) phylogenetic analyses of the SSU (16S and 18S) rRNA sequences were done using RAxML-HPC 8.2.10 (Stamatakis, 2014) at the CIPRES Portal (http://www.phylo.org/sub_sections/; Miller *et al.*, 2010) with the GTR+Γ substitution model and rapid bootstrapping. ML phylogenetic analyses of protein sequence alignments were carried out using RAxML-HPC and IQ-TREE v1.5.5 (Nguyen *et al.,* 2015) with the substitution model LG4X (Le *et al.*, 2012). In RAxML analyses, rapid bootstraping was used for Ebo proteins and nonparametric bootstrapping was used for the phylogenomic supermatrix. In IQ-TREE analyses, ultrafast bootstrapping with 1 000 replicates (Minh *et al.*, 2013) was employed for all datasets. The phylogenomic supermatrix was also analysed using Bayesian inference implemented in PhyloBayes MPI 1.7b (Lartillot *et al.*, 2013) available on the MetaCentrum VO portal (metavo.metacentrum.cz). Two independent chains were run with the CAT-GTR model for 18 000 and 24 000 iterations, respectively. Both runs converged to the same tree topology (Figure 3a) with maxdiff < 0.0057. The consensus tree was made with bpcomp by sampling every 10th tree and discarding the first 2 500 iterations as burnin. Details specific for individual phylogenetic analyses are provided in legends to respective figures. Phylogenetic trees were displayed and adjusted using iTOL (http://itol.embl.de/; Letunic and Bork, 2016), with the final graphical processing done using Inkscape 0.91 (Free Software Foundation Inc., Boston, USA). Details specific for individual phylogenetic analyses are provided in legends to respective figures.

Supplementary References

Abeel T, Van Parys T, Saeys Y, Galagan J, Van de Peer Y. (2012). GenomeView: a next-generation genome browser. *Nucleic Acids Res* **40**: e12.

Altschul SF, Madden TL, Schäffer AA, Zhang J, Zhang Z, Miller W *et al*. (1997). Gapped BLAST and PSI-BLAST: a new generation of protein database search programs. *Nucleic Acids Res* **25**: 3389–3402.

Bocs S, Cruveiller S, Vallenet D, Nuel G, Médigue C. (2003). AMIGene: Annotation of MIcrobial Genes. *Nucleic Acids Res* **31**: 3723–3726.

Castresana J. (2000). Selection of conserved blocks from multiple alignments for their use in phylogenetic analysis. *Mol Biol Evol* **17**: 540–552.

Eddy SR. (2009). A new generation of homology search tools based on probabilistic inference. *Genome Inform* **2009**: 205–211.

El Karkouri K, Kowalczewska M, Armstrong N, Azza S, Fournier PE, Raoult D. (2017). Multi-omics analysis sheds light on the evolution and the intracellular lifestyle strategies of Spotted Fever Group *Rickettsia* spp. *Front Microbiol* **8**: 1363.

Emms DM, Kelly S. (2015). OrthoFinder: solving fundamental biases in whole genome comparisons dramatically improves orthogroup inference accuracy. *Genome Biol* **16**: 157.

Fawley KP, Eliáš M, Fawley MW. (2014). The diversity and phylogeny of the commercially important algal class Eustigmatophyceae, including the new clade Goniochloridales. *J Appl Phycol* **26**: 1773–1782.

Fichant G, Basse MJ, Quentin Y. (2006). ABCdb: an online resource for ABC transporter repertories from sequenced archaeal and bacterial genomes. *FEMS Microbiol Lett* **256**: 333–339.

Finn RD, Coggill P, Eberhardt RY, Eddy SR, Mistry J, Mitchell AL *et al*. (2016). The Pfam protein families database: towards a more sustainable future. *Nucleic Acids Res* **44**: D279–D285.

Fuchs BM, Glöckner FO, Wulf J, Amann R. (2000). Unlabeled helper oligonucleotides increase the in situ accessibility to 16S rRNA of fluorescently labeled oligonucleotide probes. *Appl Environ Microbiol* **66**: 3603–3607.

Hyatt D, Chen GL, Locascio PF, Land ML, Larimer FW, Hauser LJ. (2010). Prodigal: prokaryotic gene recognition and translation initiation site identification. *BMC Bioinformatics* **11**: 119.

Lagesen K, Hallin P, Rødland EA, Staerfeldt HH, Rognes T, Ussery DW. (2007). RNAmmer: consistent and rapid annotation of ribosomal RNA genes. *Nucleic Acids Res* **35**: 3100–3108.

Letunic I, Bork P. (2016). Interactive tree of life (iTOL) v3: an online tool for the display and annotation of phylogenetic and other trees. Nucleic Acids Res **44**: W242–W245.

Lowe TM, Eddy SR. (1997). tRNAscan-SE: a program for improved detection of transfer RNA genes in genomic sequence. *Nucleic Acids Res* **25**: 955–964.

Kanehisa M, Sato Y, Kawashima M, Furumichi M, Tanabe M. (2016). KEGG as a reference resource for gene and protein annotation. *Nucleic Acids Res* **44:** D457–D462.

Katana A, Kwiatowski J, Spalik K, Zakrys B, Szalacha E, Szymanska H. (2001). Phylogenetic position of *Koliella* (Chlorophyta) as inferred from nuclear and chloroplast small subunit rDNA. *J Phycol* **37**: 443–451.

Kato K, Tanaka R, Sano S, Tanaka A, Hosaka H. (2010). Identification of a gene essential for protoporphyrinogen IX oxidase activity in the cyanobacterium *Synechocystis* sp. PCC6803. Proc Natl Acad Sci U S A **107**: 16649–16654.

Katoh K, Standley DM. (2013). MAFFT multiple sequence alignment software version 7: improvements in performance and usability. *Mol Biol Evol* **30**: 772–780.

Kim E, Harrison JW, Sudek S, Jones MD, Wilcox HM, Richards TA et al. (2011). Newly identified and diverse plastid-bearing branch on the eukaryotic tree of life. *Proc Natl Acad Sci U S A* **108**: 1496–1500.

Kück P, Meusemann K. (2010). FASconCAT: Convenient handling of data matrices. *Mol Phylogenet Evol* 56: 1115–1118.

Lagesen K, Hallin P, Rødland EA, Staerfeldt HH, Rognes T, Ussery DW. (2007). RNAmmer: consistent and rapid annotation of ribosomal RNA genes. *Nucleic Acids Res* **35**: 3100–3108.

Langmead B, Salzberg SL. (2012). Fast gapped-read alignment with Bowtie 2. *Nat Methods* **9**: 357–359.

Lartillot N, Rodrigue N, Stubbs D, Richer J. (2013). PhyloBayes MPI: phylogenetic reconstruction with infinite mixtures of profiles in a parallel environment. *Syst Biol* **62**: 611–615.

Le SQ, Dang CC, Gascuel O. (2012). Modeling protein evolution with several amino acid replacement matrices depending on site rates. *Mol Biol Evol* **29**: 2921–2936.

Letunic I, Bork P. (2016). Interactive tree of life (iTOL) v3: an online tool for the display and annotation of phylogenetic and other trees. *Nucleic Acids Res* **44**: W242–W245.

Li H, Handsaker B, Wysoker A, Fennell T, Ruan J, Homer N, Marth G, Abecasis G, Durbin R; 1000 Genome Project Data Processing Subgroup. (2009). The Sequence Alignment/Map format and SAMtools. *Bioinformatics* **25**: 2078–2079.

Miller MA, Pfeiffer W, Schwartz T. (2010). Creating the CIPRES Science Gateway for inference of large phylogenetic trees. Proceedings of the Gateway Computing Environments Workshop (GCE), New Orleans, LA, p. 1–8. (doi:10.1109/GCE.2010.5676129)

Milne I, Stephen G, Bayer M, Cock PJA, Pritchard L, Cardle L, Shaw PD, Marshall D. (2013). Using Tablet for visual exploration of second-generation sequencing data. *Brief Bioinform* **14**: 193-202.

Minh BQ, Nguyen MAT, von Haeseler A. (2013). Ultrafast approximation for phylogenetic bootstrap. *Mol Biol Evol* **30**: 1188–1195.

Nguyen LT, Schmidt HA, von Haeseler A, Minh BQ (2015). IQ-TREE: A fast and effective stochastic algorithm for estimating maximum likelihood phylogenies. *Mol Biol Evol* **32**: 268–274.

Nichols HW. (1973). Growth media – freshwater. In: Stein, J (ed). Handbook of phycological methods, culture methods and growth measurements. Cambridge University Press: Cambridge, pp 7–24.

Pruesse E, Peplies J, Glöckner FO. (2012). SINA: accurate high-throughput multiple sequence alignment of ribosomal RNA genes. *Bioinformatics* **28**: 1823–1829.

Přibyl P, Eliáš M, Cepák V, Lukavský J, Kaštánek P. (2012). Zoosporogenesis, morphology, ultrastructure, pigment composition, and phylogenetic position of *Trachydiscus minutus* (Eustigmatophyceae, Heterokontophyta). *J Phycol* **48**: 231–242.

Rutherford K, Parkhill J, Crook J, Horsnell T, Rice P, Rajandream MA *et al*. (2000). Artemis: sequence visualization and annotation. *Bioinformatics* **16**: 944–945.

Schlösser UG. (1994). SAG-Sammlung von Algenkulturen at the University of Göttingen Catalogue of strains. *Botanica Acta* **107**: 113–186.

Seemann T. (2014). Prokka: rapid prokaryotic genome annotation. *Bioinformatics* **30**: 2068–2069.

Ševčíková T, Horák A, Klimeš V, Zbránková V, Demir-Hilton E, Sudek S *et al*. (2015). Updating algal evolutionary relationships through plastid genome sequencing: did alveolate plastids emerge through endosymbiosis of an ochrophyte? *Sci Rep* **5**: 10134.

Ševčíková T, Klimeš V, Zbránková V, Strnad H, Hroudová M, Vlček Č *et al*. (2016). A comparative analysis of mitochondrial genomes in eustigmatophyte algae. *Genome Biol Evol* **8**: 705–722.

Stamatakis A. (2014). RAxML version 8: a tool for phylogenetic analysis and post-analysis of large phylogenies. *Bioinformatics* **30**: 1312–1313.

Staub R. (1961). Ernährungsphysiologische Untersuchungen an der planktonischen Blaualge *Oscillatoria rubescens* DC. *Schweiz Z Hydrol* **23:** 82–198.

Yurchenko T, Ševčíková T, Strnad H, Butenko A, Eliáš M. (2016). The plastid genome of some eustigmatophyte algae harbours a bacteria-derived six-gene cluster for biosynthesis of a novel secondary metabolite. *Open Biol* **6**: 160249.
