# Supplementary material for: A gene transfer event suggests a long-term partnership between eustigmatophyte algae and a novel lineage of endosymbiotic bacteria
Source: ISME J. 2018 Jun 7;12(9):2163–75. doi: 10.1038/s41396-018-0177-y (PMC6092422; doi:10.1038/s41396-018-0177-y)
Supplement: Supplementary file 2 — Supplementary figures [file 41396_2018_177_MOESM2_ESM.pdf]

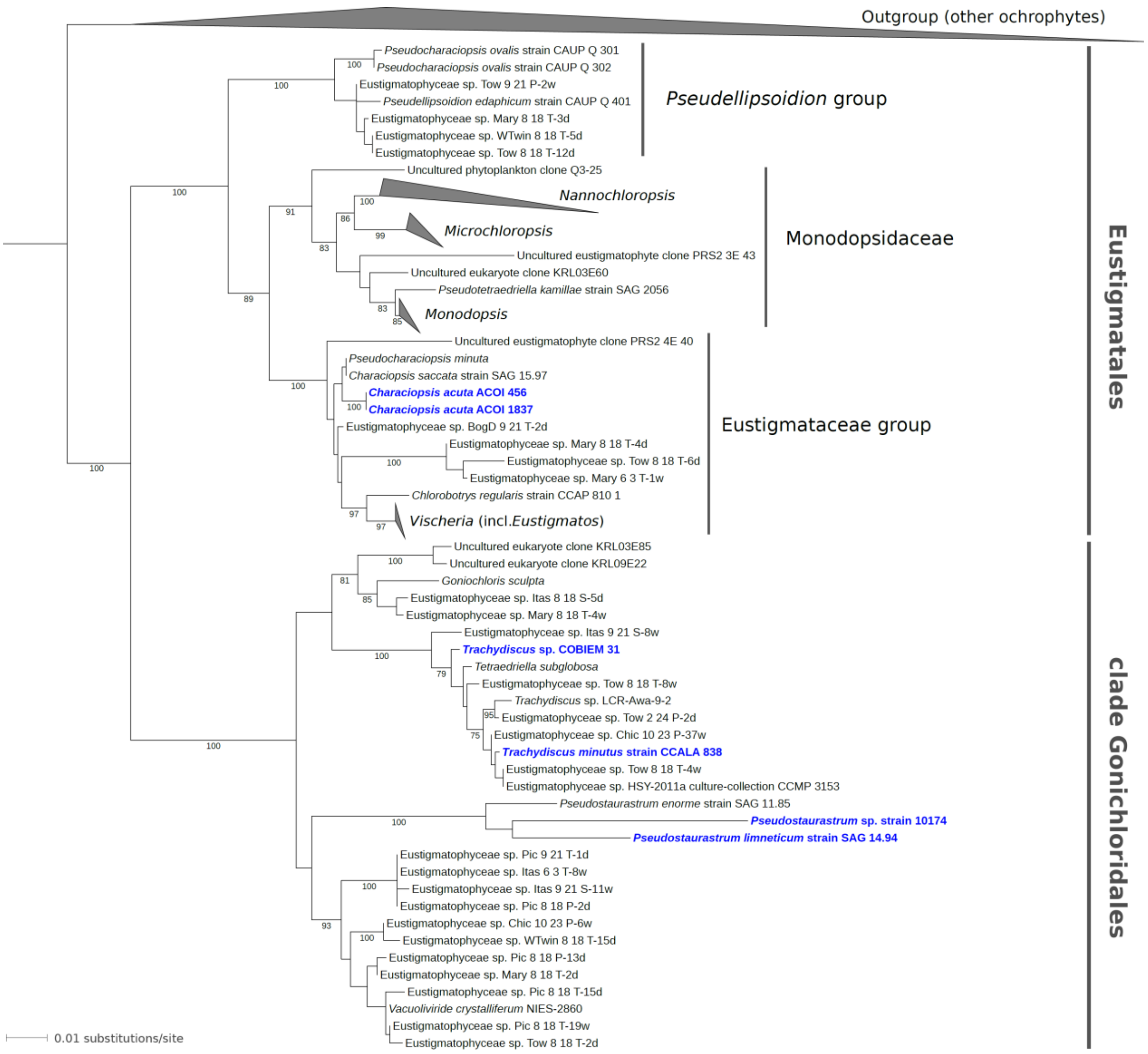


**Supplementary Figure 1** Maximum likelihood tree inferred from 18S rRNA gene sequences of eustigmatophytes and selected other ochrophytes. The tree was inferred using RAxML (GTR+$\Gamma$ substitution model). Bootstrap values are shown only when ≥75%. For simplicity, some of the clades were collapsed as triangles without showing individual constituent sequences. Eustigmatophyte strains shown to harbour *Ca.* Phycorickettsia are highlighted in blue.

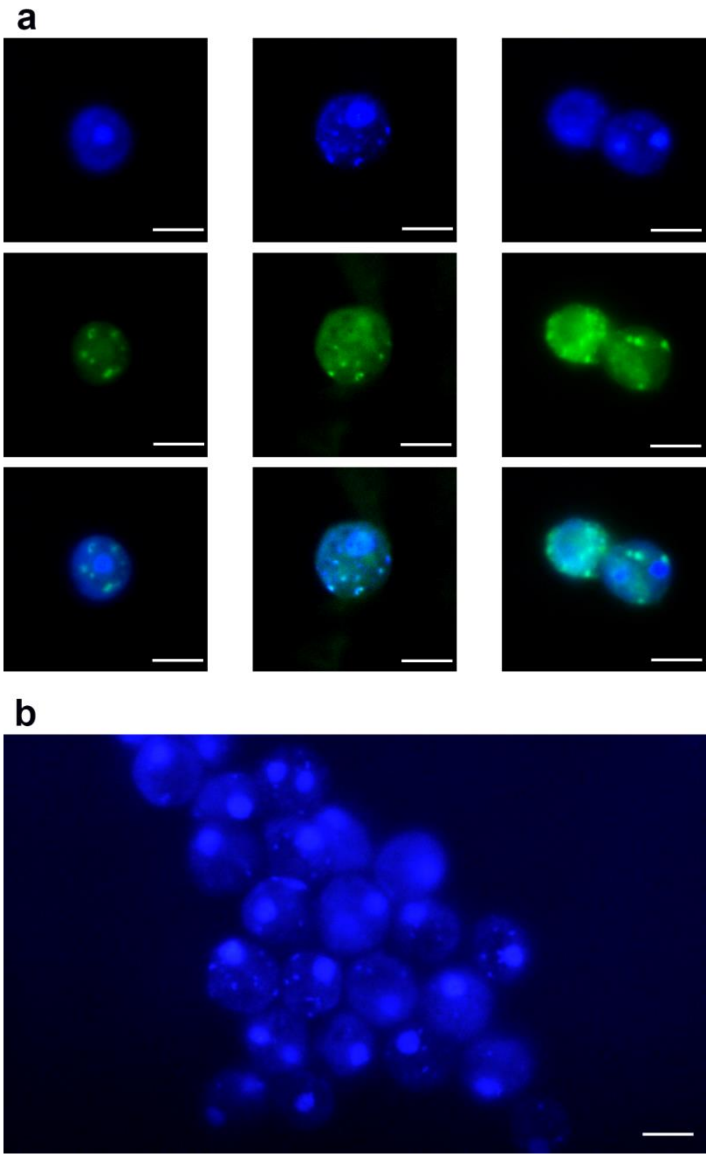

**Supplementary Figure 2** *Candidatus* Phycorickettsia trachydisci in cells of *Trachydiscus minutus* visualized by fluorescence microscopy. (**a**) Detection of *Ca.* P. trachydisci in different algal cells (each cell or cell group is shown in a separate column). First row: DAPI staining; second row: fluorescence *in situ* hybridization with the probe 16S1; third row: overlap of the signals from the first and second row. (**b**) DAPI staining of endosymbiotic bacteria in a cluster of *T. minutus* cells. Scale bar 5 µm.

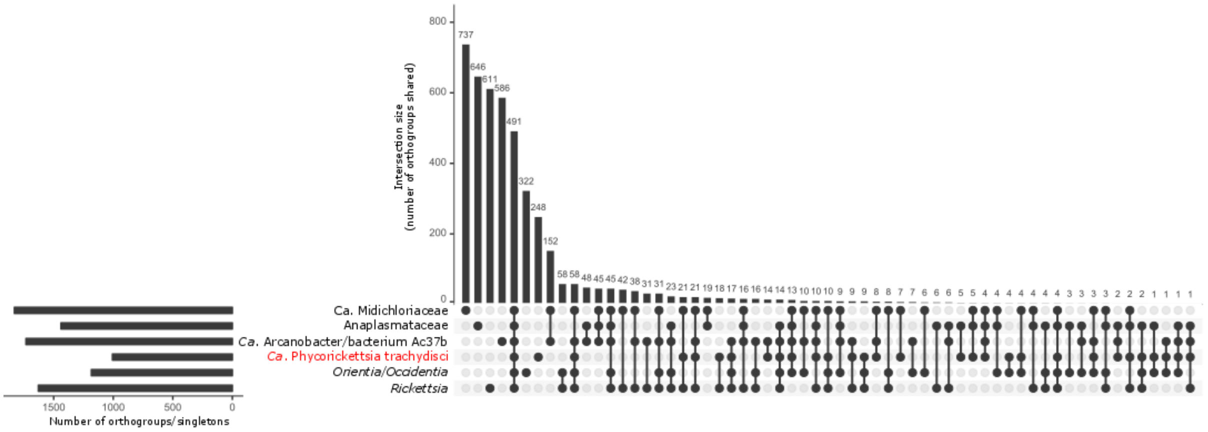


**Supplementary Figure 3** Gene sharing among *Phycorickettsia* and five other major lineages of Rickettsiales. Groups of orthologous genes (orthogroups) were defined using Orthofinder and a set of the 18 Rickettsiales species included in the tree in Figure 3a. The presence of an orthogroup in a lineage means that at least one species of the lineage has a gene of the orthogroup. Orthogroups exclusive for a particular lineage include also clusters of paralogs and singletons specific for particular species belonging to the lineage. The plot was drawn using UpSetR (Lex *et al.*, 2014).

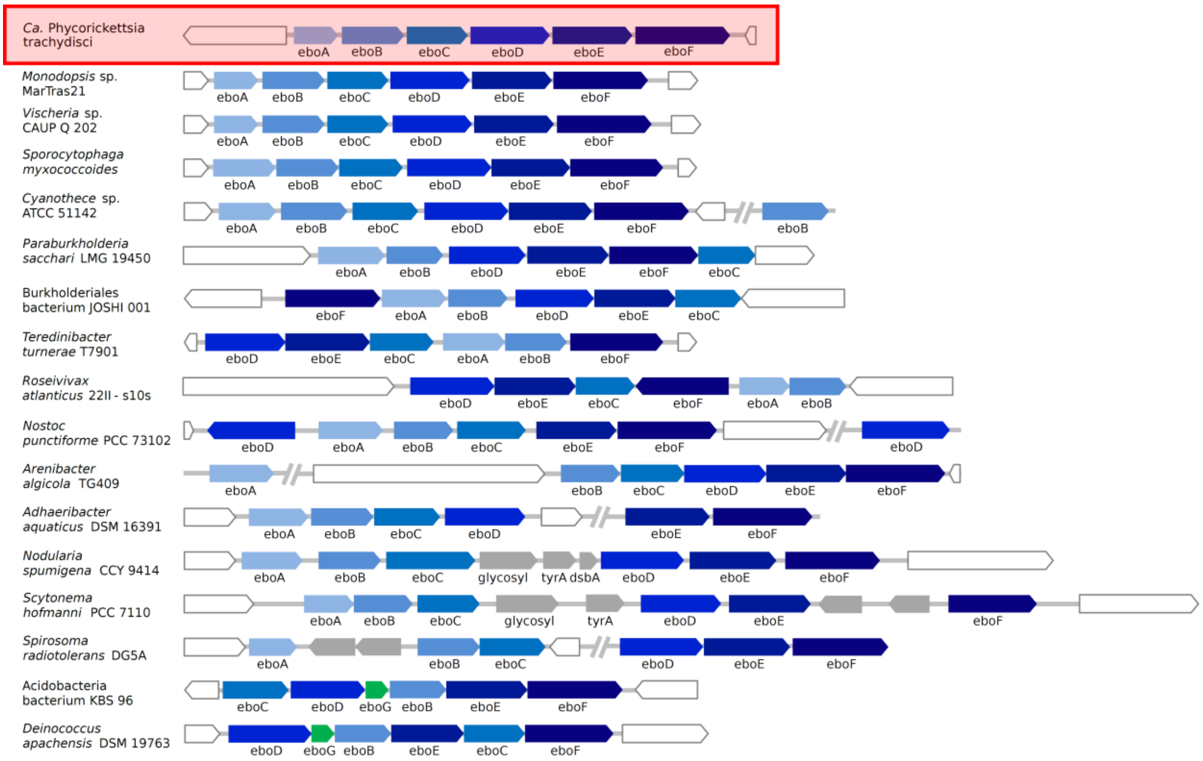


**Supplementary Figure 4** The architecture of the *ebo* operon. The figure was prepared as a modification of Figure 3 in our previous paper (Yurchenko *et al.*, 2016) by incorporation of the *ebo* operon of *Ca.* Phycorickettsia trachydisci. The scheme demonstrates the same arrangement of the *ebo* genes in *Ca.* Phycorickettsia trachydisci as in the plastid genomes of *Vischeria* sp. CAUP Q 202 and *Monodopsis* sp. MarTras21 and in some bacteria (the ABCDEF type of the operon; see Yurchenko *et al.*, 2016).

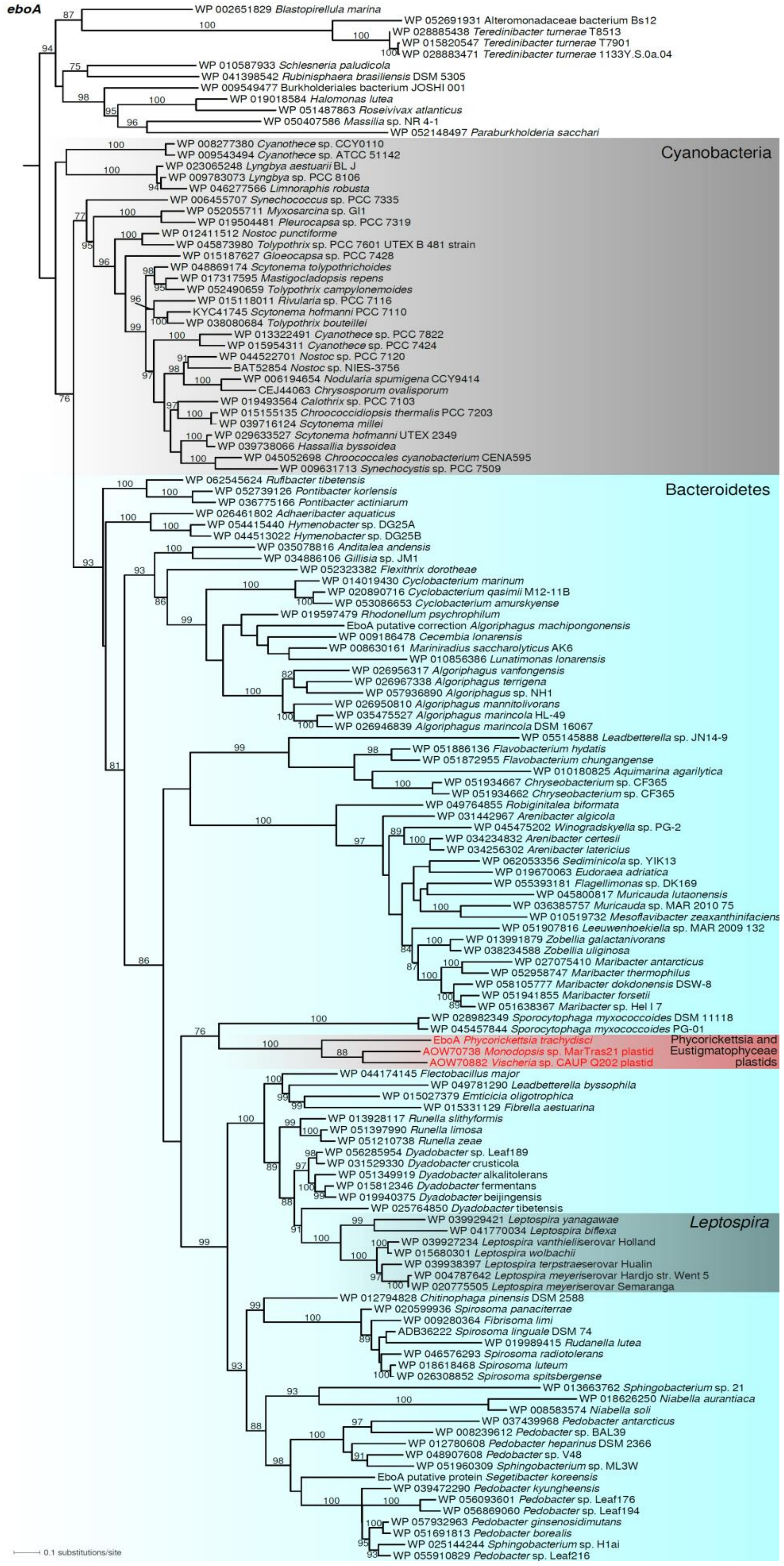


**Supplementary Figure 5** Phylogenetic analyses of Ebo proteins. Sequences from *Ca.* Phycorickettsia trachydisci were added to the alignments of their respective homologs used in similar analyses reported previously (Yurchenko *et al.*, 2016). The trees were inferred using maximum likelihood (IQ-TREE, LG4X+$\Gamma$4 substitution model). Bootstrap values (based on 1000 ultrafast bootstrap replicates) are shown only when ≥75%. For simplicity, clades comprising solely bacterial sequences were collapsed as triangles without showing individual constituent sequences. (**a**) Phylogenetic tree of EboA sequences (182 amino acid positions);

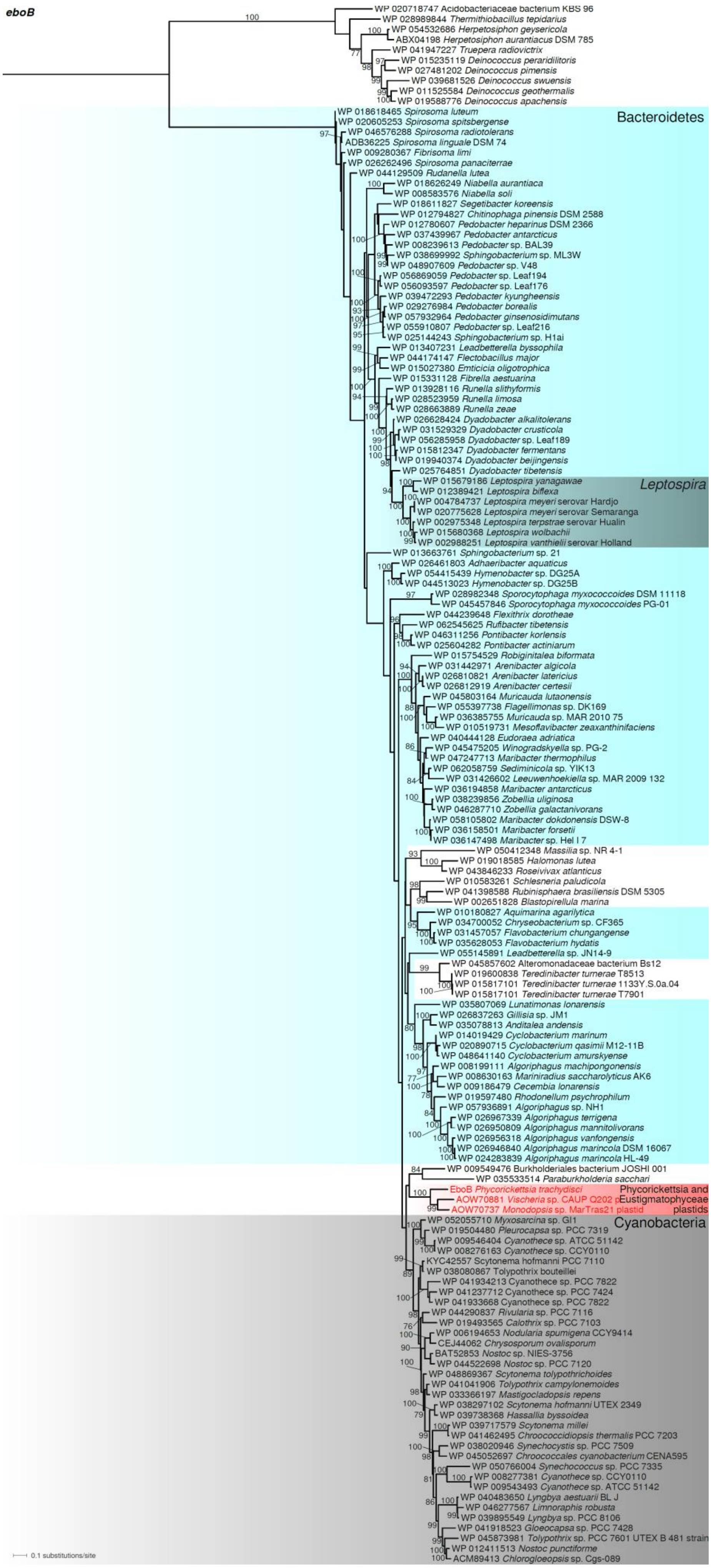

**Supplementary Figure 5** cont. (**b**) Phylogenetic tree of EboB sequences (285 amino acid positions);

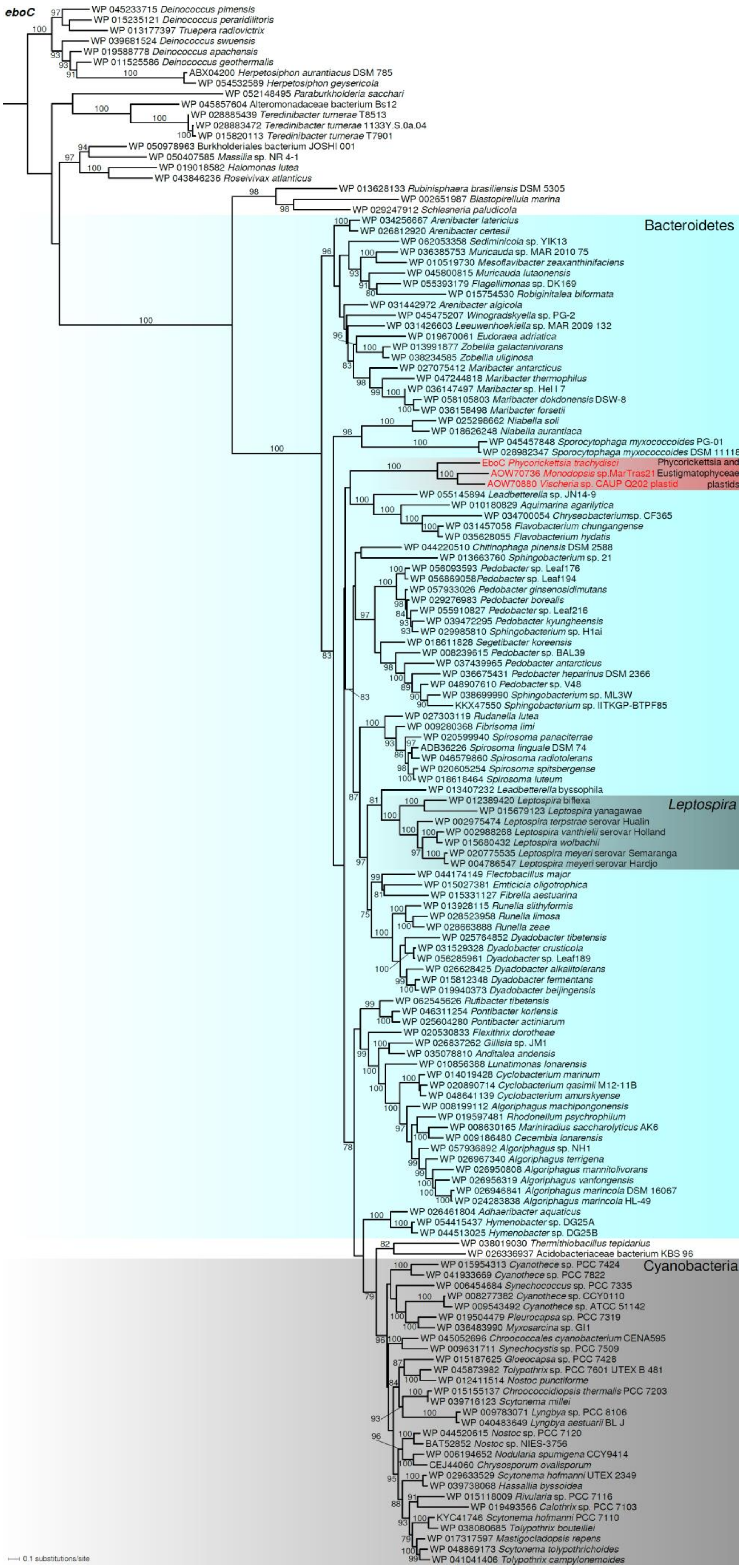


**Supplementary Figure 5** cont. (**c**) Phylogenetic tree of EboC sequences (224 amino acid positions);

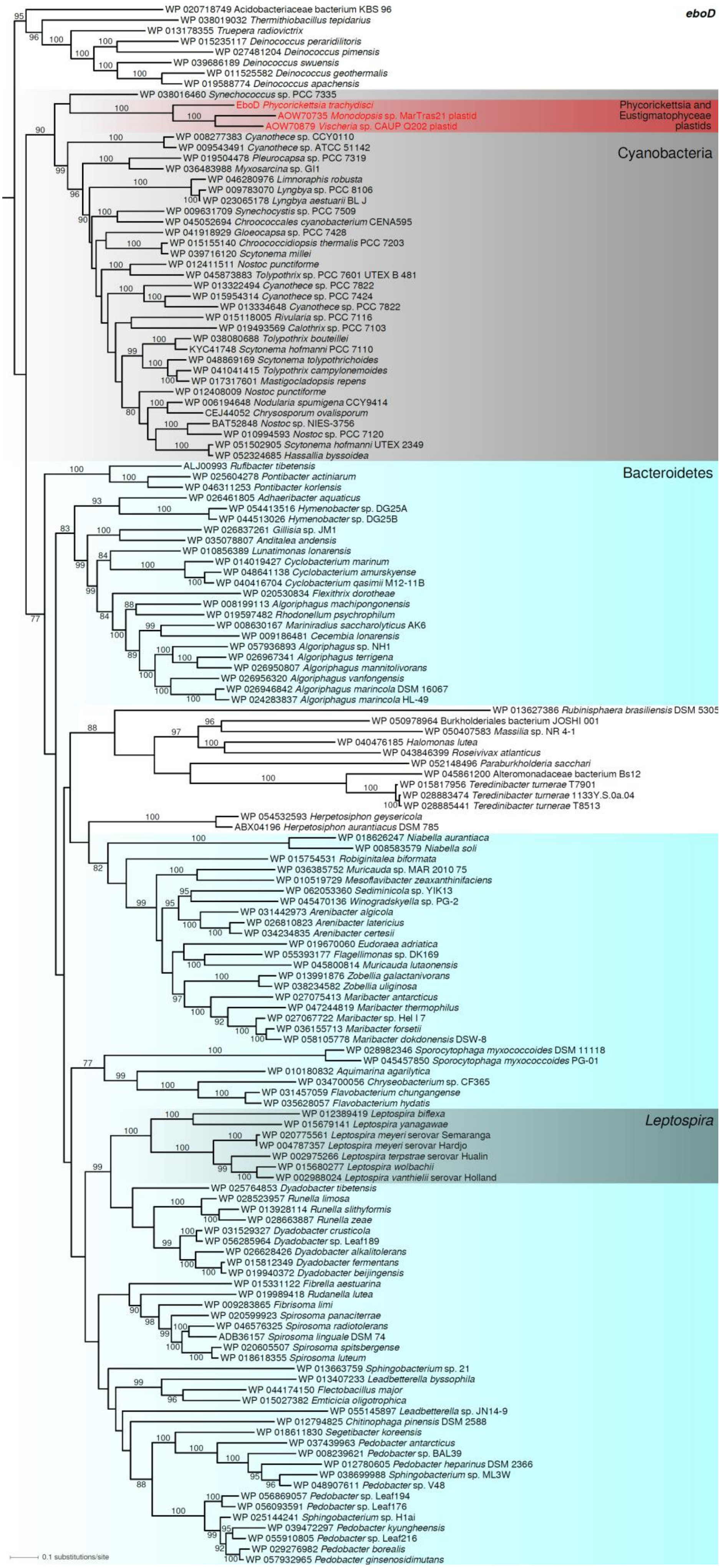


**Supplementary Figure 5** cont. (**d**) Phylogenetic tree of EboD sequences (333 amino acid positions);

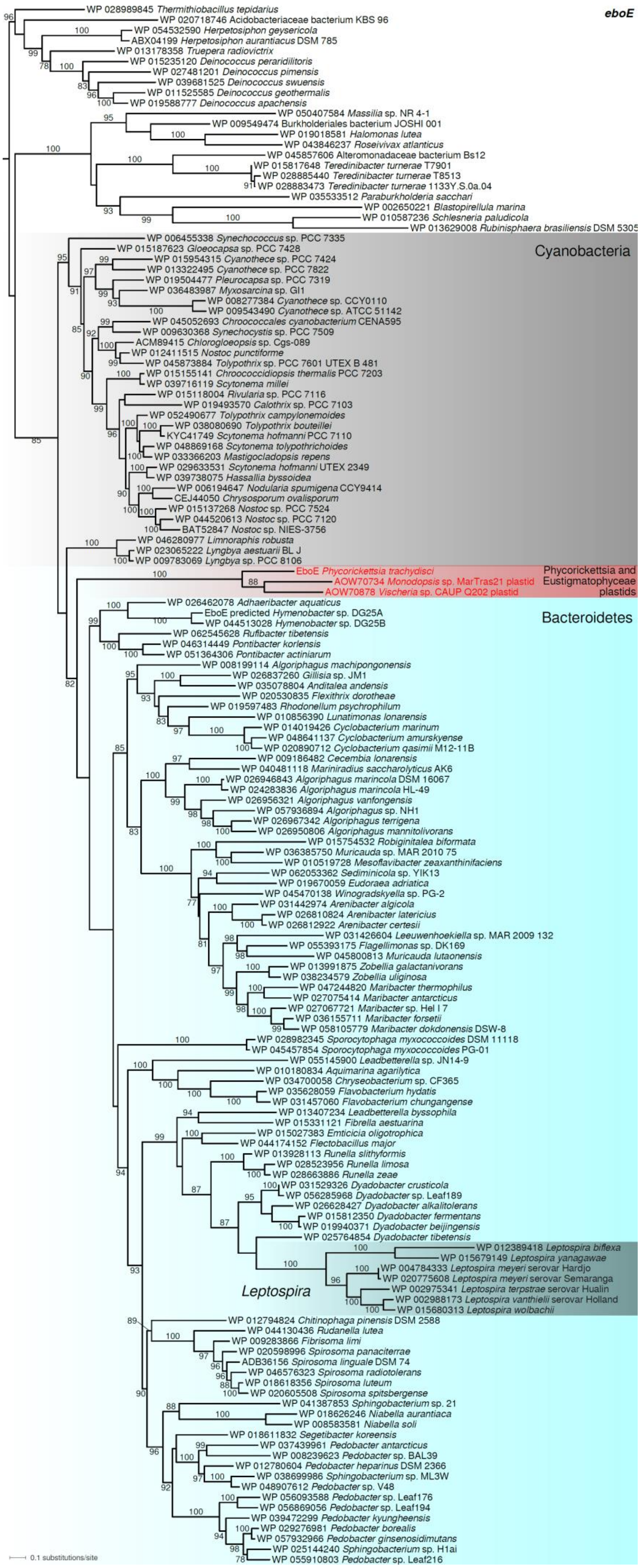


**Supplementary Figure 5** cont. (**e**) Phylogenetic tree of EboE sequences (314 amino acid positions);

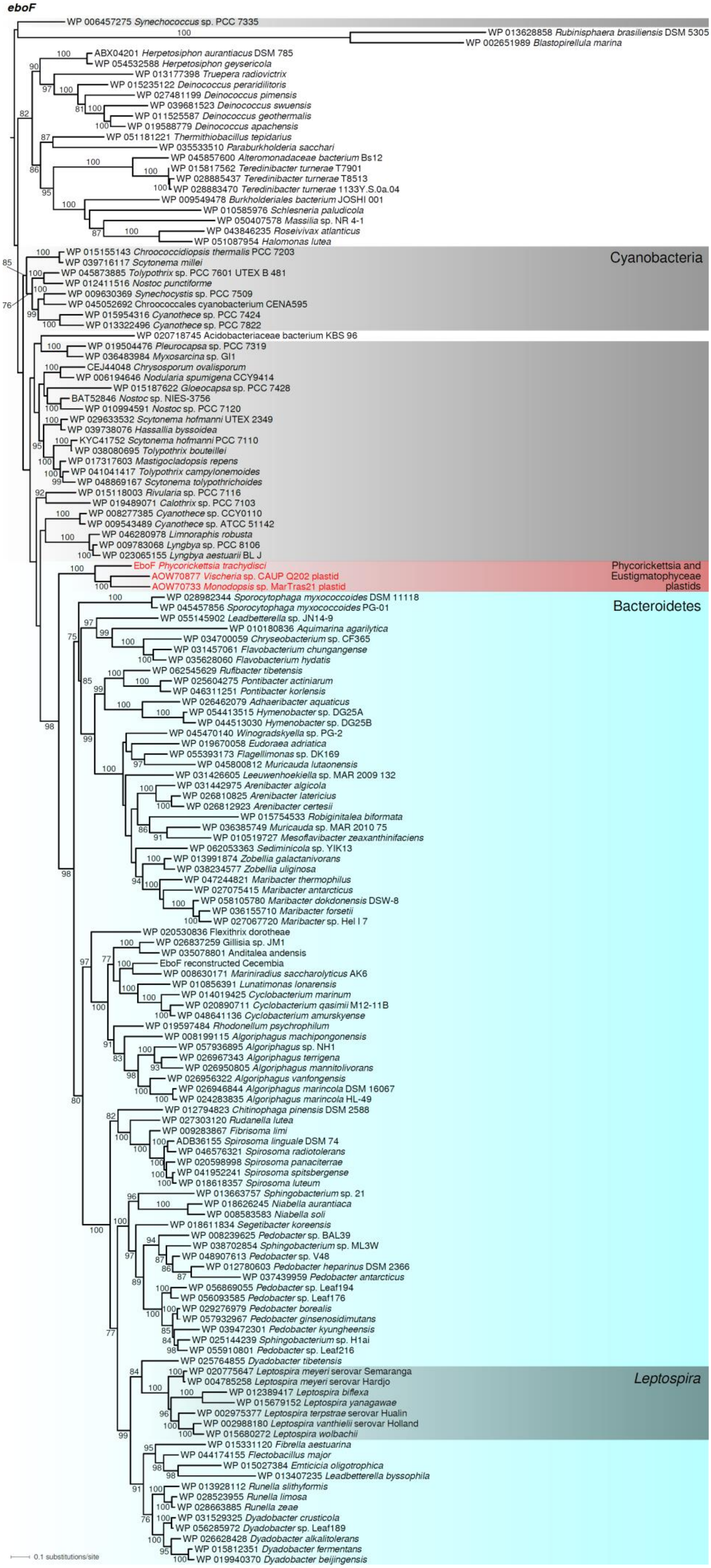


**Supplementary Figure 5** cont. (**f**) Phylogenetic tree of EboF sequences (409 amino acid positions).
