# Supplementary material for: A gene transfer event suggests a long-term partnership between eustigmatophyte algae and a novel lineage of endosymbiotic bacteria
Source: ISME J. 2018 Jun 7;12(9):2163–75. doi: 10.1038/s41396-018-0177-y (PMC6092422; doi:10.1038/s41396-018-0177-y)
Supplement: Supplementary file 3 — Supplementary Tables 1 & 2 [file 41396_2018_177_MOESM3_ESM.pdf]

Supplementary Table 1 The list of algal strains used in the study. The origin of the new isolates is specified in Supplementary Materials and Methods.

Eustigmatophyte strain	source	DNA sample	Newly sequenced 18S rDNA (GenBank accession number)
Characiopsis acuta ACOI 1837	ACOI	this study	
Characiopsis acuta ACOI 456	ACOI	this study	
Characiopsis saccata SAG 15.97	SAG	Fawley *et al.*, 2014	
Goniochloris sculpta SAG 29.96	SAG	this study	
Monodus guttula CCALA 828	CCALA	Fawley *et al.*, 2014	
Pseudostaurastrum enorme SAG 11.85	SAG	this study	
Pseudostaurastrum limneticum SAG 14.94	SAG	this study	
Pseudostaurastrum sp. strain 10174	new isolate	this study	MH045495
Pseudotetraëdriella kamillae SAG 2056	SAG	this study	
Trachydiscus minutus CCALA 838	CCALA	Ševčíková *et al.* 2015	
Trachydiscus sp. COBIEM 31	new isolate	this study	MH045496

Supplementary Table 2 The list of bacterial species used in the comparative genomic analysis. Accession numbers for the ankyrin repeat proteins counted for each genome are provided in Supplementary Table 3.

Rickettsiales strain	Genome sequence (NCBI accession number)	No. of encoded proteins	No. of ANK repeat proteins
Anaplasma marginale str. Florida	NC_012026.1	938	5
Candidatus Arcanobacter lacustris	JYHA00000000.1	882	18
Ehrlichia canis str. Jake	NC_007354.1	922	5
Candidatus Jidaibacter acanthamoeba	NZ_JSWE00000000.1	1 880	104
Candidatus Megaira subclade D (endosymbiont of *Ichthyophthirius multifiliis*)	unpublic; kindly provided by Thomas Doak	1 287	17
Candidatus Midichloria mitochondrii	NC_015722.1	1 153	12
Candidatus Phycorickettsia trachydiscis	CP027845.1	1 248	142
Neorickettsia risticii str. Illinois	NC_013009.1	759	4
Occidentia massiliensis	NZ_CANJ00000000.1	1 019	38
Orientia chuto str. Fuller	NZ_LANP00000000.1	769	30
Orientia tsutsugamushi str. Boryong	NC_009488.1	1 234	37
Rickettsia bellii RML369-C	NC_007940.1	1 356	27
Rickettsia conorii str. Malish 7	NC_003103.1	1 236	4
Rickettsia felis URRWXCal2	NC_007109.1<br>NC_007110.1<br>NC_007111.1	1 261	22
Rickettsia rickettsii str. 'Sheila Smith'	NC_009882.1	1 246	12
Rickettsia typhi str. Wilmington	NC_006142.1	816	1
Rickettsiales bacterium Ac37b	NZ_CP009217.1<br>NZ_CP009218.1<br>NZ_CP009219.1	1 639	71
Wolbachia endosymbiont of *Drosophila melanogaster*	NC_002978.6	1 024	20
